# Comprehensive Genetic Map of Muscle Lipidome Reveals Novel Insights Into Flavor Variation in Ruminant Meat

**DOI:** 10.1002/advs.202506900

**Published:** 2025-08-11

**Authors:** Xueying Zhang, Yuanyuan Kong, Jialei Chen, Ran Li, Zhongyu Wang, Yali Song, Tianshu Dai, Yuxin Fu, Beixiang Jiang, Jize Zeng, Shengwei Pei, Yangkai Liu, Qianjie Feng, Weiwei Fu, Zehu Yuan, Joram Mwashigadi Mwacharo, Fadi Li, Xiangpeng Yue

**Affiliations:** ^1^ State Key Laboratory of Herbage Improvement and Grassland Agro‐Ecosystems College of Pastoral Agriculture Science and Technology Lanzhou University Lanzhou Gansu 730000 China; ^2^ College of Animal Science and Technology Northwest A&F University Yangling Shanxi 712100 China; ^3^ Joint International Research Laboratory of Agriculture and Agri‐Product Safety of Ministry of Education Yangzhou University Yangzhou Jiangsu 225000 China; ^4^ Dryland Livestock Genomics International Centre for Agricultural Research in the Dry Areas (ICARDA) Addis Ababa 5689 Ethiopia; ^5^ Animal and Veterinary Sciences Scotland's Rural College Roslin Institute Building Edinburgh EH25 9RG Scotland

**Keywords:** genomics, lipidomics, meat flavor, mGWAS, sheep

## Abstract

Ruminant meat is an important component of human diets, valued for its unique flavor and nutritional density. Lipids play a dominant role in shaping meat flavor, yet their genetic and biochemical basis remains unexplored. Here, from the analysis of 434 sheep *longissimus thoracis* samples, the current study presents the first comprehensive lipid map of sheep meat, including 947 lipids. A substantial proportion of these lipids exhibit moderate‐to‐high heritability, with 51.6% surpassing a heritability of 0.2 and 15.8% exceeding 0.45. Metabolome‐based genome‐wide association analysis identifies 467 significant loci affecting 233 lipids, including 110 loci exhibiting pleiotropy. Notably, the levels of monogalactosyldiacylglycerols containing oleic (C18:1) and linoleic (C18:2) acids are specifically regulated by the expression of *MBOAT1* and *PAQR8* genes, respectively, while 13 triglycerides and one diglyceride are co‐regulated by *SH2D4A*. The levels of phosphatidylethanolamine PE(20:4_20:0) are regulated by *VPS53*. Further examination of volatile compounds demonstrates that variations in these genetically controlled lipids significantly impact flavourant levels in cooked meat. Given the conservation of lipid profiles and genomes among ruminants, this study offers novel insights into the genetic architecture underlying meat lipid metabolism and provides a valuable resource for the targeted genetic improvement of ruminant meat flavor.

## Graphical abstract

Ruminant meat is widely appreciated for its distinctive flavor. Lipids play a central role in influencing meat flavor, yet their genetic and biochemical basis remains largely unexplored. Here, a comprehensive lipid genetic map of sheep muscle is constructed and 20 candidate genes potentially modulating lipid levels are identified to provide a roadmap to improve meat flavor.

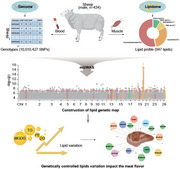

## 1 Introduction

Meat flavor is a critical indicator of meat quality and serves as the most influential factor guiding consumer preferences.^[^ ^1^, ^2^ ^]^ Ruminant meat exhibits unique flavors that distinguishes it from other meat,^[^ ^3^ ^]^ which is fundamentally attributed to its divergent composition of flavor precursors.^[^ ^4^, ^5^ ^]^ The flavor precursors in muscle mainly comprise of lipids and hydrophilic compounds, generating aromatic flavor compounds through lipid oxidation, Maillard reactions, Strecker degradation etc.^[^ ^6^, ^7^ ^]^ Notably, lipids contribute to the majority of volatile flavourants through thermochemical oxidation,^[^ ^7^, ^8^, ^9^ ^]^ particularly forming species‐specific lipid oxidation products during high‐temperature cooking (grilling and roasting) that establishes the core flavor distinction between ruminant and non‐ruminant meats.^[^ ^10^ ^]^ However, the challenges in large‐scale sampling after slaughter have limited systematic studies of the lipid profile of ruminant muscle thereby restricting further research and improvement in meat flavor.

In the past few decades, genomic studies have provided valuable insights into the domestication events and some traits formation of ruminants,^[^ ^11^ ^]^ but their meat flavor traits have remained poorly investigated. In particular, the unclear genetic architecture underlying ruminant meat lipid profiles poses a significant barrier to genetic improvement of flavor traits. Metabolome‐based genome‐wide association study (mGWAS) is a powerful tool for analyzing associations between a wide range of lipids and genetic variants to reveal the genetic basis of metabolic diversity,^[^ ^12^ ^]^ and has been used to identify genetic factors influencing fruit flavor in plants.^[^ ^13^, ^14^ ^]^ However, due to challenges and difficulties of phenotypic measurements and the greater complexity of flavor‐regulating mechanisms in livestock compared to plants, mGWAS research on meat flavor remain scarce. This knowledge gap limits our understanding of lipid heritability, major‐effect genes, and related quantitative trait loci (QTLs) in livestock meat. So far, only duck has been identified several QTLs affecting the levels of metabolites and volatiles associated with meat flavor and quality traits using the mGWAS method,^[^ ^15^ ^]^ demonstrating the potential of mGWAS strategies to elucidate the genetic regulators of flavor‐related lipids in livestock.

Here, we employed comprehensive lipid profiling and mGWAS to uncover the intricate genetic mechanisms underlying lipid metabolism in ruminant meat, using sheep as the study model. By integrating lipidomic and volatile compound data, we assessed the impact of lipid composition on meat flavor. Our findings present the first comprehensive lipid genetic map of ruminant meat, marking the beginning of an exploration into the biochemical foundations and genetic architecture shaping ruminant meat flavor.

## 2 Results

### 2.1 A comprehensive Lipid Map of *Longissimus Thoracis* (LT) Muscle

**Figure 1:**
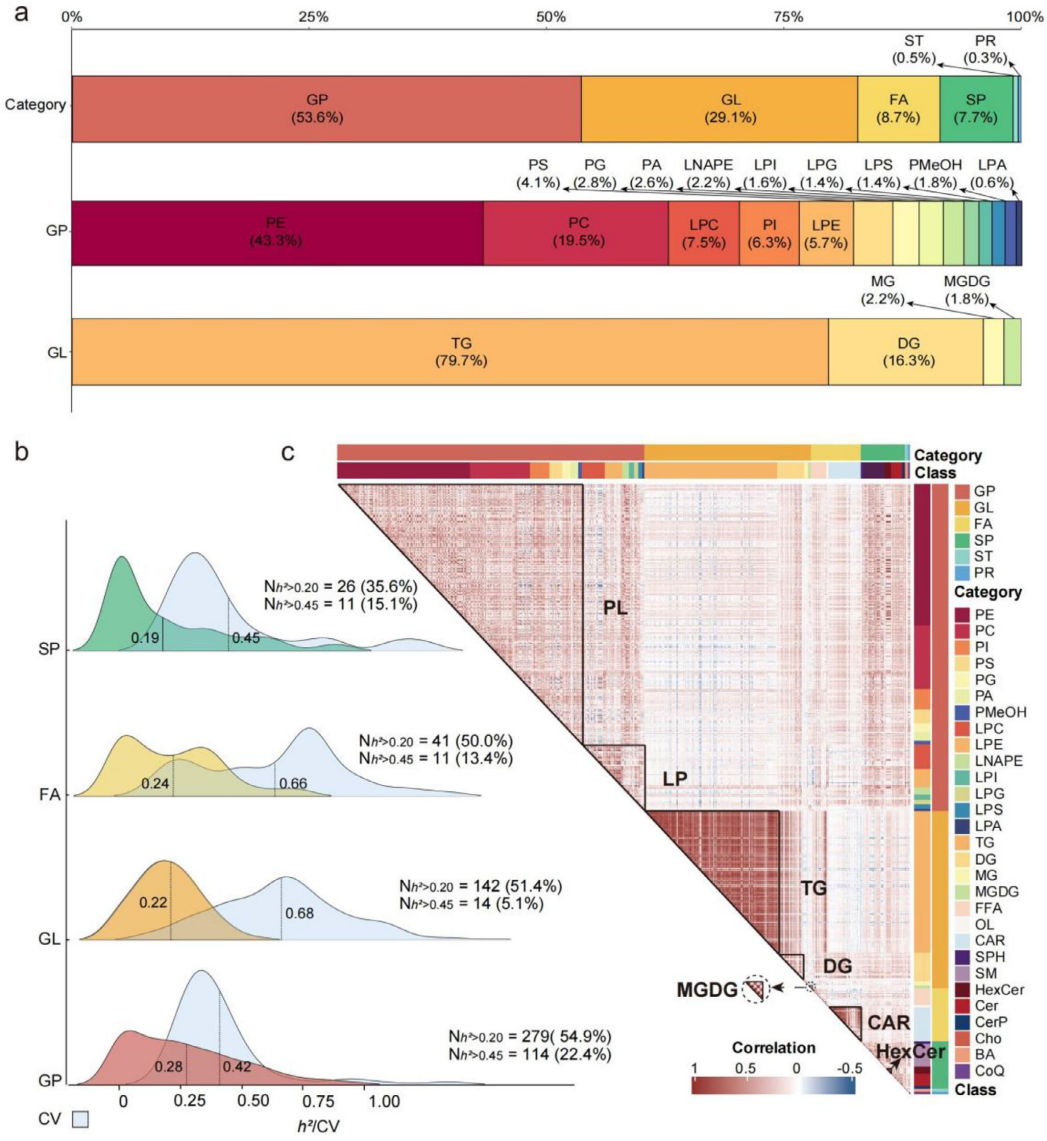
Lipidomic profiling of *longissimus thoracis* in Hu sheep. a) Lipid species composition and distribution. A stacked bar chart shows the numerical proportion of lipids within each category/class. The 947 lipids belong to six categories, with the GP and GL accounting for the highest proportion. b) Heritability (*h^2^*) and coefficient of variation (CV) of 947 lipids. The *h^2^* is colour‐coded according to four lipid categories (GP, GL, FA, and SP), while ST and PR are not displayed due to insufficient lipid numbers. The CV is shown in light blue. Vertical dashed lines indicate the average values. c) Heatmap displaying pairwise Spearman correlations between lipid species included in the clusters with the strongest correlations. The clusters are marked by black lines and labeled by the included lipid classes. GL: glycerolipid, GP: glycerophospholipid, FA: fatty acyl, ST: sterol lipid, SP: sphingolipid, PR: prenol lipid, TG: triglyceride, DG: diglyceride, MG: monoglyceride, MGDG: monogalactosyldiacylglycerol, PS: phosphatidylserine, PI: phosphatidylinositol, PG: phosphatidylglycerol, PE: phosphatidylethanolamine, PC: phosphatidylcholine, PA: phosphatidic acid, PMeOH: Phosphatidyl methanol, LP: lysophosphatide, LNAPE: N‐acyl‐lysophosphatidyl ethanolamine, LPA: lysophosphatidic acid, LPC: lysophosphatidylcholine, LPE: lysophosphatidylethanolamine, LPG: lysophosphatidylglycerol, LPI: lysophosphatidylinositol, LPS: lysophosphatidylserine, BA: bile acid, CAR: carnitine, Cer: ceramide, Cho: cholesterol, CoQ: coenzyme Q, OL: oxidized lipid, FFA: free fatty acid, HexCer: hexosylceramide, SM: sphingomyelin, SPH: Sphingosine.

A comprehensive lipid map from 434 sheep LT muscles was established using the widely targeted lipidomics approach. A total of 947 lipid species belonging to 35 classes were identified to cover six major lipid categories: glycerophospholipids (GP), glycerolipids (GL), fatty acyls (FA), sphingolipids (SP), sterol lipids (ST), and prenol lipids (PR), which accounted for 53.6%, 29.1%, 8.7%, 7.7%, 0.5% and 0.3% of the total lipids, respectively (**Figure** **1**a; Table S1, Supporting Information). In particular, phosphatidylethanolamines (PE) and phosphatidylcholines (PC) dominate in the GP category, representing 43.3% and 19.5%, respectively. In the GL category, triglycerides (TG) were the most abundant, accounting for 79.7% (Figure 1a).

The coefficient of variation (CV) ranged from 9.92% for MG(16:0) to 173.48% for taurocholic acid, with 80.57% of the lipids exhibiting CVs > 30% (Figure 1b; Figure S1, Table S1, Supporting Information). This substantial phenotypic variability highlights that lipids have significant potential for breeding improvement. The estimation of heritability (*h^2^*) showed that GPs had the highest average *h^2^* (0.28), followed by FAs (0.24), and GLs (0.22) (Figure 1b; Table S2, Supporting Information). Notably, 51.6% of the lipids displayed moderate‐to‐high heritability (*h^2^* > 0.20), and 15.8% displayed high heritability (*h^2^* > 0.45), primarily in GPs (114/508) and GLs (14/276), suggesting that genetic factors significantly influence the levels of flavor‐related lipids.

The pairwise Spearman correlation analyses revealed that the strongest correlations were observed within the same lipid class (Figure 1c; Table S3, Supporting Information). Three distinct correlation clusters emerged within the GL category: TG, diacylglycerols (DG), and monogalactosyldiacylglycerols (MGDG), where TG and MGDG clusters demonstrated particularly strong associations (*r* > 0.8). In the GP category, lysophospholipids (LP) and phospholipids (PL) formed two separate correlation clusters. Additionally, a clear positive correlation was also noted among carnitines (CAR) within the FA category and hexosylceramides (HexCer) within the SP category. These results indicate that lipids of the same class are likely to be regulated by the same metabolic pathways.

Given the predominance of GP and GL as major lipid categories in lamb meat (Figure 1a), we constructed extreme phenotype models stratified on the basis of total GP/GL levels (Figure S2, Supporting Information) to investigate their differential impacts on cooked meat flavor. Gas chromatography‐ion mobility spectrometry (GC‐IMS) identified 62 volatile compounds (Table S4, Supporting Information), which were then subjected to partial least‐squares discriminant analysis (PLS‐DA) modeling for group discrimination (GP_H/GP_L and GL_H/GL_L; H and L denoting high and low, respectively) using a machine learning algorithm. Notably, the GP‐based model demonstrated superior explanatory power, accounting for 62.5% of flavor variance compared to 51.8% for GL (Figure S3a, Supporting Information). This differential predictive capacity was supported by confusion matrix analyses, with GP groups achieving 97.5% classification accuracy versus 89.0% for GL (Table S5, Supporting Information). Further comparison of volatile compound abundances revealed category‐specific modulation patterns: GP significantly increased the total levels of alcohols while reducing the levels of ketones, whereas GL significantly elevated the total levels of esters (Figure S3b,c, Supporting Information). Twelve and five differentially abundant volatile compounds were identified between the GP_H and GP_L groups, and the GL_H and GL_L groups, respectively (Figure S3d,e, Supporting Information). Taken together, these results indicate that GPs have a relatively greater impact on meat flavor than GLs.

### 2.2 Genetic Basis of Lipids in LT Muscle

**Figure 2:**
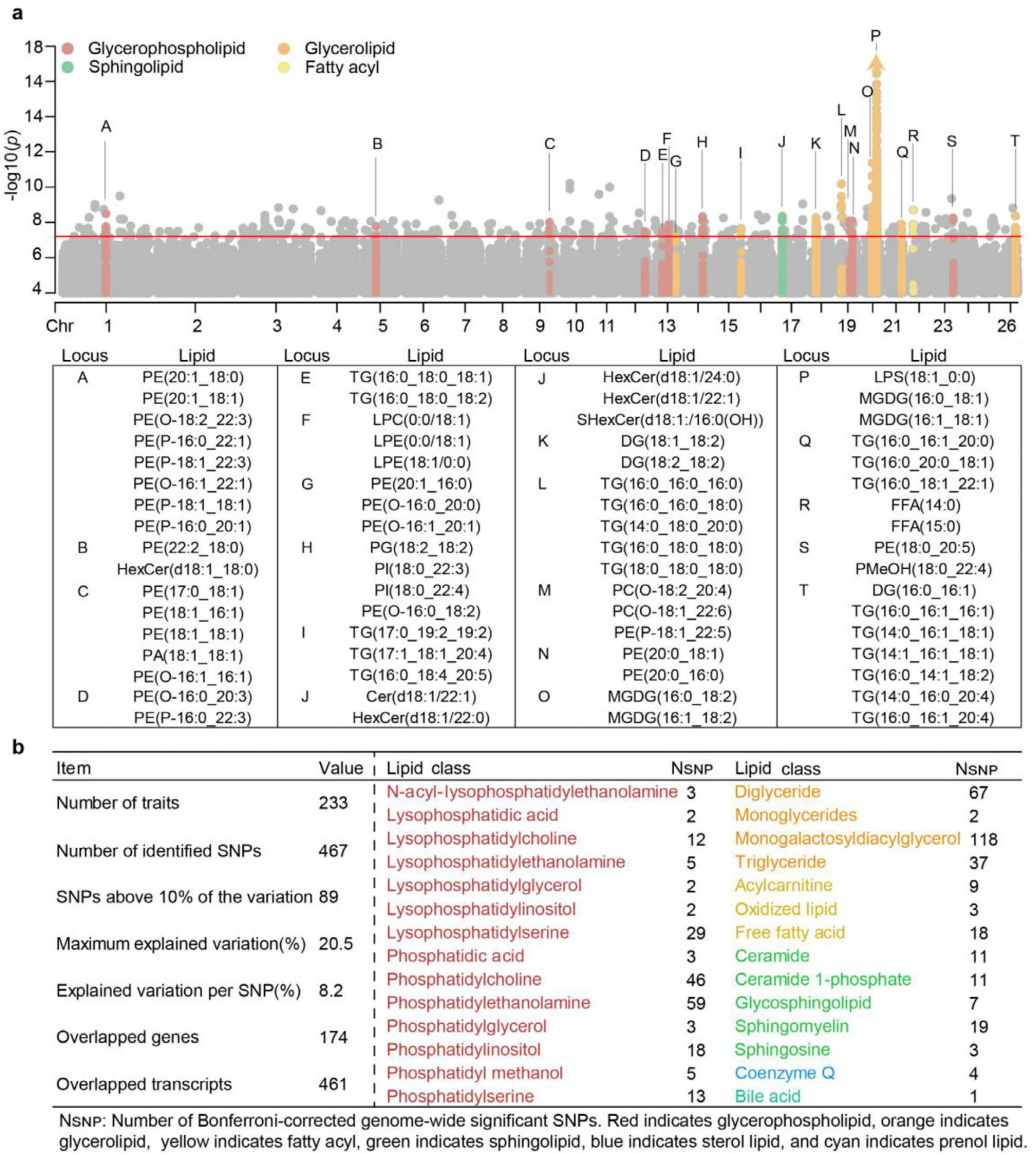
Summary of genetic loci associated with lipid levels. a) Manhattan plot of mGWAS. Replicated signals are labeled with uppercase letters and mapped to genomic positions (detailed in the table). Genome‐wide significance thresholds are indicated by a red horizontal line (*P* < 6.26 × 10^−8^), and extreme associations (*P* < 1.0 × 10^−18^) are marked with triangles. b) Summary of significant lipid‐SNP associations identified in mGWAS.

We performed whole‐genome sequencing for 434 individuals with a mean coverage depth of 7 ×. A total of 10010427 SNPs were identified to conduct mGWAS with 947 lipids based on a mixed linear model (MLM). A Bonferroni correction of *P* = 6.26 × 10^−8^ was employed as the genome‐wide threshold for all trait associations, identifying 467 significant SNPs associated with at least one of the 233 lipids (Table S6, Supporting Information). Manhattan plots display pleiotropic signals significantly associated with multiple lipids, including 20 signals corresponding to GP, GL, SP, and FA (**Figure** **2**a). Summary statistics for all GWAS associations are shown in Figure 2b. Overall, 24.6% of the detected lipids (233/947) exhibited at least one significant association, with an average of 2.7 associations per lipid (Figure S4, Supporting Information). These variants showed large effects when explaining the variation; for instance, of up to 20.5% for MGDG(16:0_18:1), and an average of 8.2% (Table S6, Supporting Information). The correlation between the variance explained and heritability was 0.47. GLs were associated with most SNPs (224/467), with the MGDGs (118/224) and DGs (67/224) accounting for the highest proportion. Based on the mGWAS results and gene expression levels, we identified 20 candidate genes modulating lipid levels (**Table** **1**). These genes and their associated lipids were co‐enriched in glycerolipid, glycerophospholipid, and inositol phosphate metabolism pathways, revealing their potential regulatory roles in lipid metabolism (Figure S5, Supporting Information). Interestingly, the lipids associated within the same signal were also from the same correlation cluster shown in Figure 1c, suggesting pleiotropic effects of key metabolic enzymes in lipid metabolic pathways. In summary, we identified a large set of mGWAS‐identified genetic signals related to muscle lipids, whose levels are subject to complex genetic regulation.

**Table 1:** Summary of 20 candidate genes assigned from mGWAS results.

SNP	Lead Lipid	*P* Value	Candidate Gene	Description
Chr1:149641958	PE(P‐18:1_18:1)	4.26 × 10^−8^	*CHODL*	Chondrolectin
Chr1:282674515	FFA(19:0)	3.28 × 10^−8^	*BRWD1*	Bromodomain and WD Repeat Domain Containing 1
Chr3:82421632	TG(8:0_16:1_18:1)	1.81 × 10^−8^	*SOCS5*	Suppressor Of Cytokine Signaling 5
Chr3:230068316	PS(17:0_20:4)	5.16 × 10^−8^	*PEX26*	Peroxisomal Biogenesis Factor 26
Chr4:121822157	FFA(14:0)	1.06 × 10^−8^	*ZNF786*	Zinc Finger Protein 786
Chr7:54861681	LPC(28:1)	2.50 × 10^−8^	*MNS1*	Meiosis Specific Nuclear Structural 1
Chr10:37311079	DG(16:1_18:0)	6.07 × 10^−8^	*FGF9*	Fibroblast Growth Factor 9
Chr10:95786354	HexCer(d18:1/18:0)	4.40 × 10^−8^	*SOX1*	SRY‐Box Transcription Factor 1
Chr11:41288499	PE(20:4_20:0)	1.01 × 10^−10^	*VPS53*	Vacuolar Protein Sorting‐Associated Protein 53 Homolog
Chr13:24659965	LPE(18:1_0:0)	4.00 × 10^−8^	*PIP4K2A*	Phosphatidylinositol‐5‐Phosphate 4‐Kinase Type 2 Alpha
Chr14:14049756	LPG(18:1_0:0)	1.75 × 10^−8^	*ZNF469*	Zinc Finger Protein 469
Chr14:51804122	SM(d18:1/23:0)	2.35 × 10^−8^	*GMFG*	Glia Maturation Factor Gamma
Chr17:18641620	LPC(17:0/0:0)	2.86 × 10^−8^	*RNF150*	Ring finger protein 150
Chr17:79860092	PC(16:0_22:6)	3.80 × 10^−8^	*PISD*	Phosphatidylserine Decarboxylase
Chr20:26347961	MGDG(16:0_18:2)	4.21 × 10^−12^	*PAQR8*	Progestin and AdipoQ Receptor Family Member 8
Chr20:41026667	MGDG(16:0_18:1)	2.35 × 10^−23^	*MBOAT1*	Membrane Bound O‐Acyltransferase Domain Containing 1
Chr21:46456337	TG(16:0_18:4_20:5)	3.62 × 10^−8^	*NDUFV1*	NADH:Ubiquinone Oxidoreductase Core Subunit V1
Chr22:20868113	Cer(d18:2_23:0)	4.82 × 10^−8^	*PGAM1*	Phosphoglycerate Mutase 1
Chr23:24783079	LPC(0:0_18:0)	1.07 × 10^−8^	*FHOD3*	Formin Homology 2 Domain Containing 3
Chr26: 41738166	TG(14:0_16:0_20:4)	4.43 × 10^−9^	*SH2D4A*	SH2 Domain‐Containing Protein 4A

### 2.3 Genetic Variations Associated with MGDG and One Substrate Lysophosphatidylserine (LPS)

MGDGs contribute to characteristic flavors by degrading unsaturated fatty acid chains on their carbon skeleton to produce flavor compounds during heating.^[^ ^16^, ^17^ ^]^ Interestingly, we discovered that two of the strongest mGWAS signals are associated with four MGDGs (MGDG(16:0_18:1), MGDG(16:1_18:1), MGDG(16:0_18:2), MGDG(16:1_18:2)) and one substrate LPS(18:1_0:0) of MGDG containing a C18:1 branch on chromosome 20.

**Figure 3:**
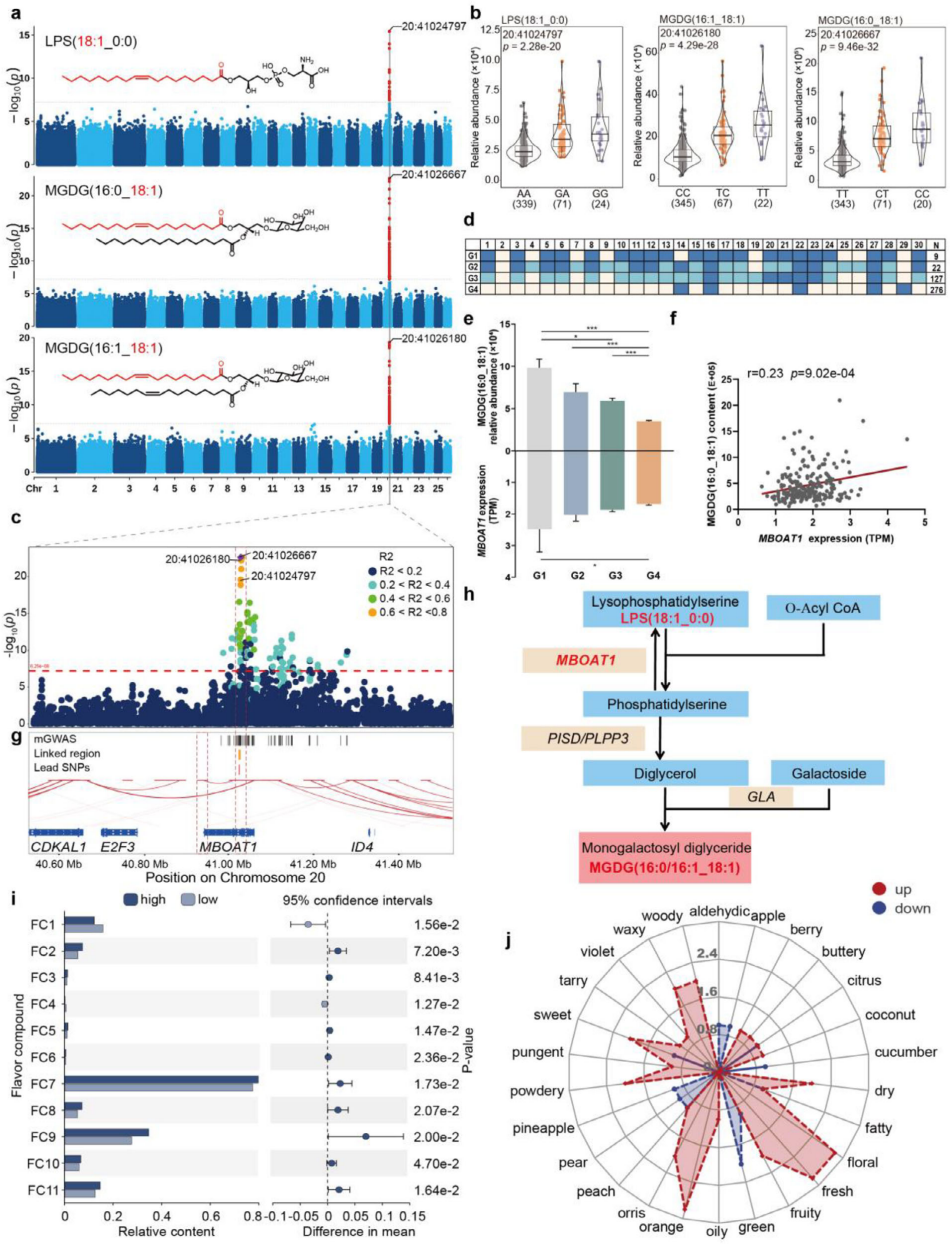
Genetic regulations of LPS(18:1_0:0), MGDG(16:0_18:1), and MGDG(16:1_18:1) and their effect on meat flavor. a) Structure and mGWAS Manhattan plots for the three lipids. The horizontal grey dashed line indicates the genome‐wide significance (*P* < 6.26 × 10^−8^) threshold. b) Comparison of the lipid abundance of different genotypes of the lead SNPs (Chr20: 41024,797 bp, Chr20: 41026,667 bp, Chr20: 41026,180 bp, respectively). The number of individuals are given in brackets. c) Regional locuszoom plot displaying LD patterns between the lead SNP and neighboring SNPs across the 40.53–41.53 Mbp genomic region, derived from the GWAS for MGDG(16:0_18:1) level. d) Genotype analysis was conducted based on 30 SNPs located in regions with strong linkage. 1–30 represents the 30 SNPs within the 41.02 to 41.03 Mbp region. Bases within the dark blue frame represent homozygous variants, those within the light blue frame represent heterozygous variants, and those within the white frame represent homozygous reference. e) Relative abundance of MGDG(16:0_18:1) and expression levels of *MBOAT1* across the four genotypes, data represent the mean ± SEM. *P <0.05, *** P <0.001. f) Correlation between MGDG(16:0_18:1) abundance and *MBOAT1* expression in 210 individuals. g) Plot of the identified enhancer‐promoter loop between the highly linked SNP region and *MBOAT1* promoter. Horizontal red lines show the region of loop anchors throughout the plotting area. h) The potential metabolic pathways regulated by *MBOAT1* for MGDG and LPS. i) Comparison of relative contents of key flavor compounds (rOAV > 1) between high‐ (n = 19) and low‐lipid individuals (n = 20). FC1: 3‐methyl‐butanoic acid‐butyl ester, FC2: 1‐decanol, FC3: 4‐(2,6,6‐trimethylcyclohexa‐1,3‐dienyl)but‐3‐en‐2‐one, FC4: (*E*)‐2‐nonenal, FC5: 2‐undecenal, FC6: (*E*)‐2‐undecenal, FC7: 2‐methyl‐3‐pentanol, FC8: 5‐hexyldihydro‐2(3H)‐furanone, FC9: beta‐ionone, FC10: naphthalene, FC11: alpha‐irone. j) Radar chart of flavor characteristics response from 11 flavor compounds.

The first signal, located in Chr20: 40.98‐41.28 Mbp region, was associated with MGDG(16:0_18:1), MGDG(16:1_18:1), and LPS(18:1_0:0), all of which exhibit strong positive genetic correlations with each other (*r_g_* > 0.6, **Figure** **3**a; Figures S6, S7, Supporting Information). Three lead SNPs (Chr20: 41026,667 bp, Chr20: 41026,180 bp, and Chr20: 41024,797 bp) with the highest association with MGDG(16:0_18:1), MGDG(16:1_18:1), and LPS(18:1_0:0) levels explained 20.5%, 20.1%, and 17.9% of the total variance, respectively (Figure 3b). These three SNPs were also conserved across ruminant species (Figure S8, Supporting Information). To narrow the candidate region, we calculated pairwise LD coefficients within the QTL (Chr20: 40.53‐41.53 Mbp), revealing that the SNPs located in the membrane bound O‐acyltransferase domain containing 1 (*MBOAT1*) gene were highly linked (pairwise *r^2^* > 0.6, Figure 3c). This region was classified into four combined genotypes (Figure 3d; Figure S9a, Supporting Information), among which G1 showed significantly greater lipid abundance compared to G3 and G4 (Figure 3e; Figure S9b, Supporting Information). We then compared the expression levels of all four candidate genes within a 1 Mbp region surrounding the lead SNP (Chr20: 41026,667 bp); only the expression of *MBOAT1* showed significant differences among the different genotypes (Figure 3e; Figure S9c, Supporting Information), and its expression was positively correlated (*P* < 0.05) with the levels of three lipids (Figure 3f; Figure S10, Supporting Information), suggesting that *MBOAT1* in LT muscle positively regulates the levels of MGDG(16:0_18:1), MGDG(16:1_18:1), and LPS(18:1_0:0). High‐resolution chromatin conformation capture (Hi‐C) reveals the highly linked SNPs are located within a significant enhancer‐promoter interaction loop connected to *MBOAT1* promoter (Figure 3g), which implies that alleles within this region may influence *MBOAT1* transcription by disrupting enhancer‐promoter interactions. *MBOAT1* encodes a C18:1‐preferring LPS acyltransferase^[^ ^18^ ^]^ that catalyzes the conversion of LPS(18:1_0:0) to phosphatidylserine (C18:1), which subsequently generates MGDG(C18:1) through the GP metabolism pathway (Figure 3h).

To further evaluate how variations in MGDG(16:0_18:1), MGDG(16:1_18:1), and LPS(18:1_0:0) affect meat flavor, we performed gas chromatography‐mass spectrometry (GC‐MS)‐based volatile compound profiling on cooked meat from extreme lipid abundance groups. Notably, 72 of 342 identified volatile compounds (21.1%) exhibited significant differences in abundance corresponding to variations in the three lipids (Table S7, Supporting Information). Among these, 11 compounds with relative odour activity values (rOAV) > 1 played a substantive role in meat flavor variation (Figure 3i). Nine compounds displayed elevated levels and rOAV values in high‐lipid groups, enhancing floral, fresh, orange, dry, orris, and woody flavors, while two flavor compounds (3‐methyl‐butanoic acid‐butyl ester and (*E*)‐2‐nonenal) were depleted, suppressing green, pear, pineapple, aldehydic, and cucumber flavors (Figure 3j; Figure S11, Supporting Information).

**Figure 4:**
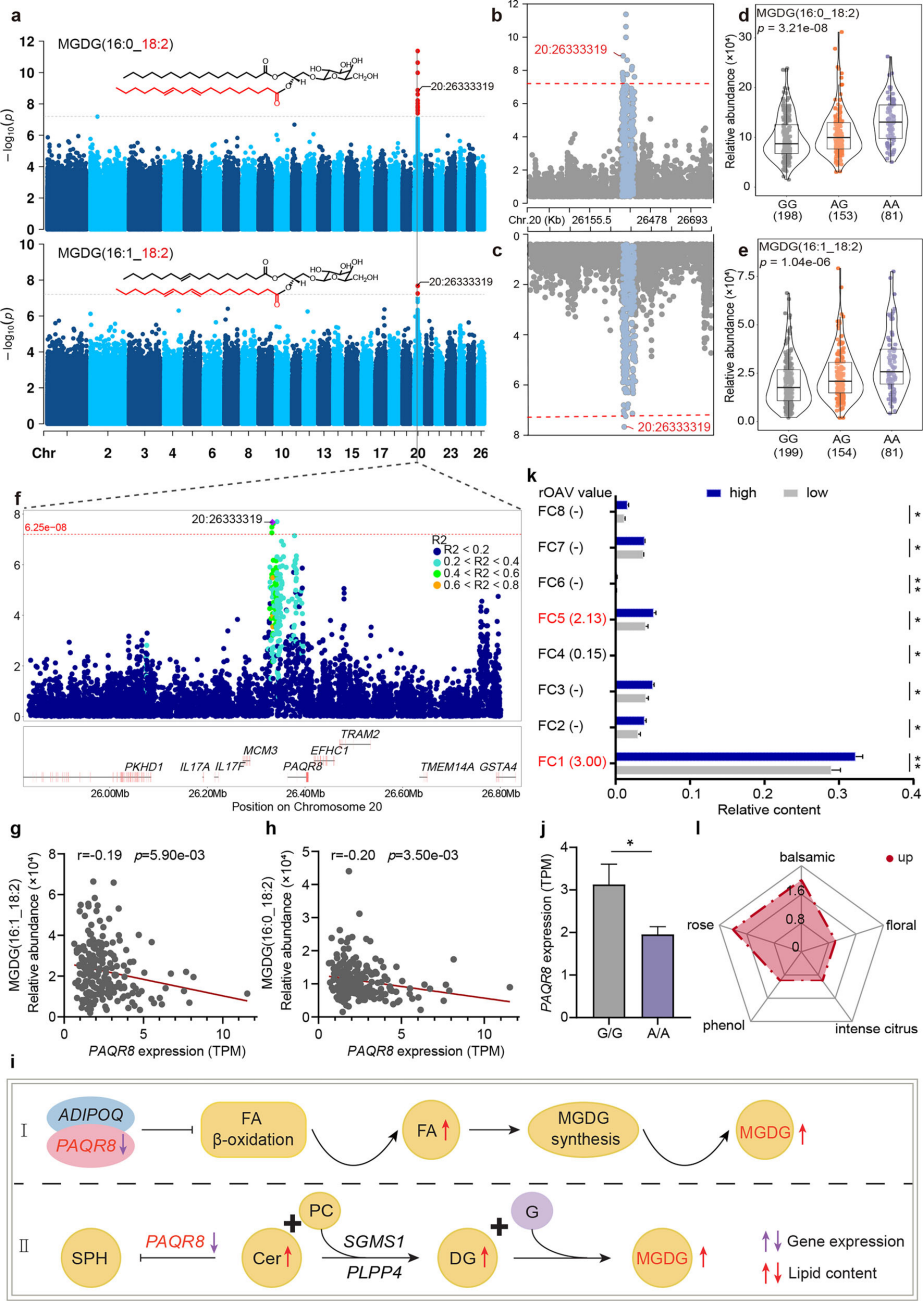
Genetic regulations of the levels of MGDG containing linoleic acid (C18:2). a) The structure and mGWAS Manhattan plot for MGDG(16:0_18:2) and MGDG(16:1_18:2). (b,c) Magnified view of the region of interest. (d,e) Comparison of the lipid abundance of different genotypes of the SNP on Chr20: 26333,319 bp. f) Regional locuszoom plot displaying the LD pattern between the Chr20: 26333,319 bp SNP and the other SNPs within the 25.83 to 26.82 Mbp region. (g,h) Correlations between MGDG(16:1_18:2) and MGDG(16:0_18:2) abundance and *PAQR8* expression (n = 210). i) The hypothesized model of *PAQR8* regulating the two MGDGs levels. FA: fatty acid, SPH: sphingosine, Cer: ceramide, PC: phosphatidylcholine, DG: diacylglycerol, G: galactoside. j) Comparison of *PAQR8* gene expression between GG and AA genotype individuals (n = 10). Data represent as mean ± SEM. *P<0.05 k) Relative content and rOAV values of differentially volatile compounds (n = 20). FC1: benzyl alcohol, FC2: 4‐allyl‐1,6‐heptadiene‐4‐ol, FC3: 2,4,4,6‐tetramethyl‐hept‐2‐ene, FC4: decanoic acid methyl ester, FC5: 1‐octanol, FC6: 2,6‐dimethyl‐6‐(4‐methyl‐3‐pentenyl)‐bicyclo[3.1.1]hept‐2‐ene, FC7: 1,3‐dimethyl‐1H‐pyrazole‐4‐carbaldehyde, FC8: 1,2,4,5‐tetrazin‐3‐amine. Only FC1 and FC5 with rOAV value >1. Data represent as mean ± SEM. *P<0.05, **P<0.01. (l) Radar chart of flavor characteristics response of two flavor compounds (FC1 and FC5).

The second signal was localized to the Chr20: 26.33‐26.39 Mbp region and associated with MGDG(16:0_18:2) and MGDG(16:1_18:2) (**Figure** **4**a; Figure S12, Supporting Information). Intriguingly, a SNP at Chr20: 26333,319 bp was linked to both MGDGs (Figure 4b,c), explaining 8.14% of the total variance in their abundance (Figure 4d,e). We examined linkage disequilibrium (LD) within a 1 Mbp region surrounding this SNP, identifying five highly linked SNPs (pairwise *r^2^* > 0.6) within 1 Kbp of the intergenic region (Figure 4f). Subsequently, we investigated the genes within a 1 Mbp region surrounding the SNP to assess their correlation with lipid abundance. Only the expression levels of progestin and adipoQ receptor family member 8 (*PAQR8*) exhibited significant negative correlations with the abundance of MGDG(16:1_18:2) (r = ‐0.19, *P* = 5.90e‐03) and MGDG(16:0_18:2) (r = −0.20, *P* = 3.50e‐03) (Figure 4g,h; Figure S13a, Supporting Information), indicating that *PAQR8* negatively regulates the levels of these two lipids in LT muscle. *PAQR8* belongs to the progestin and adipoQ receptor family and possesses both adiponectin receptor and ceramidase activity, ^[^ ^19^, ^20^ ^]^ consistent with its role in negatively regulating MGDG biosynthesis (Figure 4i). Comparison of *PAQR8* expression levels in individuals with extreme lipid levels revealed higher expression in GG individuals compared to AA individuals (Figure 4j). To investigate whether the Chr20: 26333,319 variation modulates gene expression through proximal or distal regulatory mechanisms, we employed ATAC‐seq, H3K27ac profiling, Hi‐C, and luciferase reporter assays (Figure S13b,c, Supporting Information), however, these approaches failed to establish a definitive regulatory evidence chain, warranting further investigation.

To further evaluate the impact of variations in MGDG(16:0_18:2) and MGDG(16:1_18:2) on cooked meat flavor, we conducted a GC‐MS analysis on LT samples from individuals with extreme lipid abundance. The results revealed that eight volatile compounds had significantly higher content in the group with the high lipid abundance than the low group (Figure 4k; Table S8, Supporting Information). Notably, benzyl alcohol and 1‐octanol, with rOAV > 1, contributed to the enhancement of rose, phenolic, balsamic, intense citrus, and floral flavor characteristics in sheep meat (Figure 4k,l).

### 2.4 *SH2D4A* Negatively Regulates TGs and DG Content to Enhance Meat Flavor

**Figure 5:**
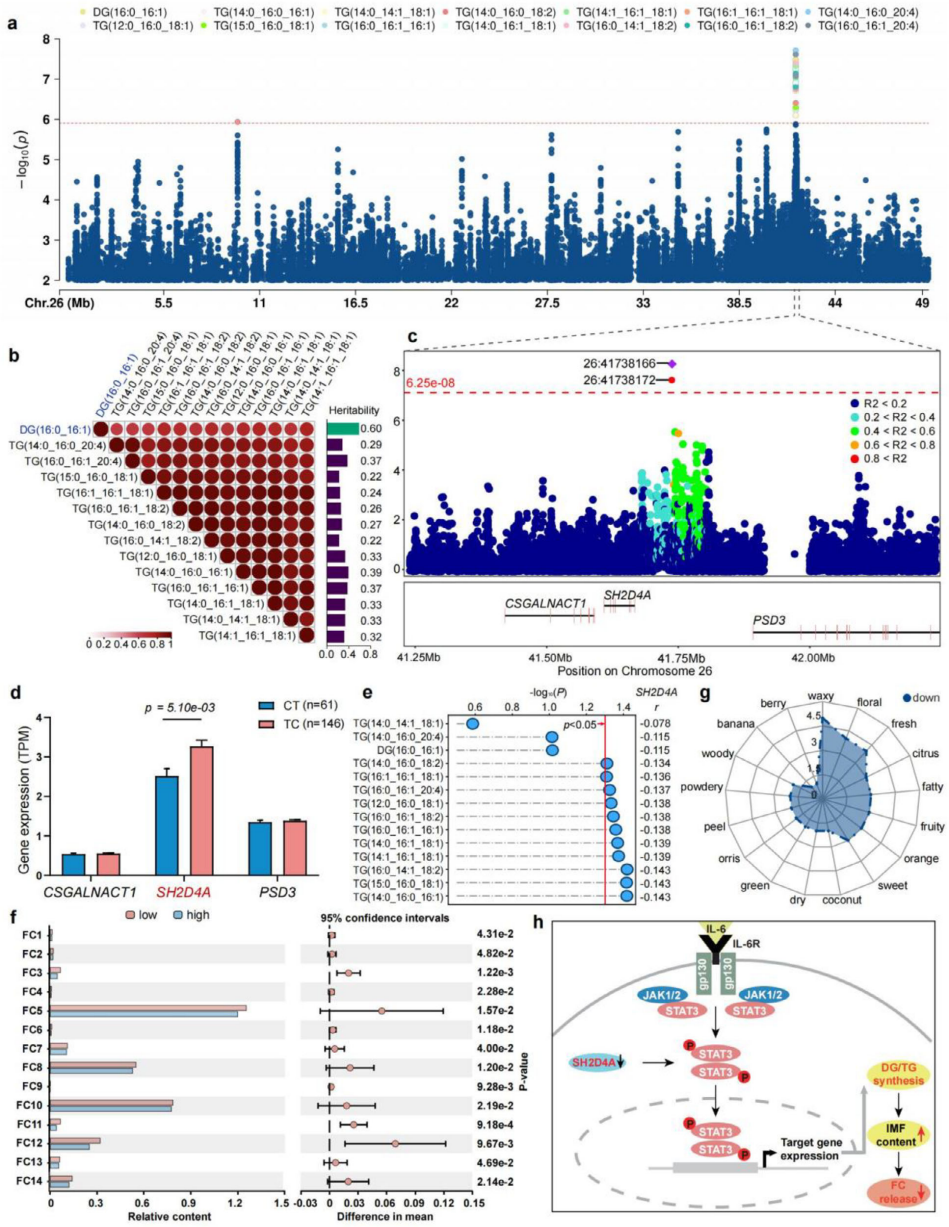
Identification of shared variants regulating TGs and DG levels and flavor quality. a) mGWAS Manhattan plots for 13 TGs and one DG, the horizontal red dashed line indicates the genome‐wide significance (*P* < 1.25 × 10^−6^) threshold. b) Genetic parameter estimates for lipids. c) Regional plots for the QTL ranging from 41.24 to 42.24 Mbp. All genotyped SNPs are color‐coded according to their pairwise LD calculated based on the lead SNP. d) Comparison of gene expressions between the two haplotypes, the data represent the mean ± SEM. The numbers of individuals are given in brackets. e) Correlations between lipids and *SH2D4A* expression in 210 individuals. f) Relative content of differentially flavor compounds with rOAV value > 1. FC1: geraniol, FC2: dodecanoic acid methyl ester, FC3: 1‐decanol, FC4: 4‐(2,6,6‐Trimethylcyclohexa‐1,3‐dienyl)but‐3‐en‐2‐one, FC5: (*E*)‐2‐octen‐1‐ol, FC6: 2‐undecenal, FC7: (*Z*)‐2‐decenal, FC8: (*E*)‐2‐octenal, FC9: (*E*)‐2‐undecenal, FC10: 2‐methyl‐3‐pentanol, FC11: 5‐hexyldihydro‐2(3H)‐furanone, FC12: beta‐ionone, FC13: naphthalene, FC14: alpha‐irone. g) Radar chart of the flavor characteristics response from the flavor compounds. h) Depiction of the hypothesis that *SH2D4A* regulates 14 lipids affecting meat flavor.

TGs and DGs are the primary components of intramuscular fat (IMF), mediating meat flavor changes in response to IMF variation.^[^ ^21^, ^22^ ^]^ One diglyceride (DG(16:0_16:1)) and 13 TGs, including TG(12:0_16:0_18:1), TG(14:0_16:0_16:1), TG(15:0_16:0_18:1), TG(14:0_14:1_18:1), TG(16:0_16:1_16:1), TG(14:0_16:0_18:2), TG(14:0_16:1_18:1), TG(14:1_16:1_18:1), TG(16:0_14:1_18:2), TG(16:1_16:1_18:1), TG(16:0_16:1_18:2), TG(14:0_16:0_20:4), TG(16:0_16:1_20:4), were controlled by a common signal on chromosome 26 and shared two SNPs (Chr26: 41738,166 bp and Chr26: 41738,172 bp) (**Figure** **5**a; Figure S14–S16, Supporting Information). These lipids have *h^2^* > 0.2 and show significant positive genetic correlations with each other (*r_g_* > 0.6), with genetic correlations greater than 0.65 within 13 TGs (Figure 5b; Table S9, Supporting Information). LD analysis showed that the two shared SNPs were in complete linkage (pairwise *r^2^* = 1, Figure 5c), and individuals with the CT haplotype exhibited significantly higher TGs and DG abundances than those with the TC haplotype (Figure S17, Supporting Information). To further identify the potential candidate gene, we compared the expression levels of three genes (*CSGALNACT1*, *SH2D4A*, *PSD3*) within a 1 Mbp region around the two SNPs, with *SH2D4A* expression showing a significant difference (Figure 5d). Correlation analysis between gene expression and lipid abundance indicated that 11 lipids were significantly negatively correlated with *SH2D4A* (|r| > 0.13, *P* < 0.05, Figure 5e). This suggests that the downregulation of *SH2D4A* releases its inhibition on *STAT3* nuclear translocation, allowing phosphorylated STAT3 to activate lipid metabolism genes, driving TG/DG synthesis (Figure 5h).^[^ ^23^, ^24^ ^]^

To evaluate how TG and DG variations influence meat flavor, we compared volatile compounds in LT muscles between extreme lipid groups. Comparative analysis revealed 117 differentially abundant volatile compounds, including 14 sensorially impactful compounds (rOAV > 1; Table S10, Supporting Information), paradoxically enriched in low‐lipid muscle (Figure 5f; Figure S18, Supporting Information). Mechanistically, while 13 TGs and 1 DG showed strong genetic‐phenotypic covariation with IMF deposition (Figure S19 and Table S11, Supporting Information), their accumulation suppressed the release of flavor compounds during cooking,^[^ ^25^ ^]^ diminishing key aromas (waxy, floral, orange, and fatty) in lipid‐rich meat (Figure 5g,h). Our results indicated that a class of lipids in the same metabolic pathways are controlled by a few large‐effect loci in the skeletal muscle metabolome, potentially influencing further the flavor of meat.

### 2.5 Identification of Synthesis‐Controlling Genes for PE(20:4_20:0)

**Figure 6:**
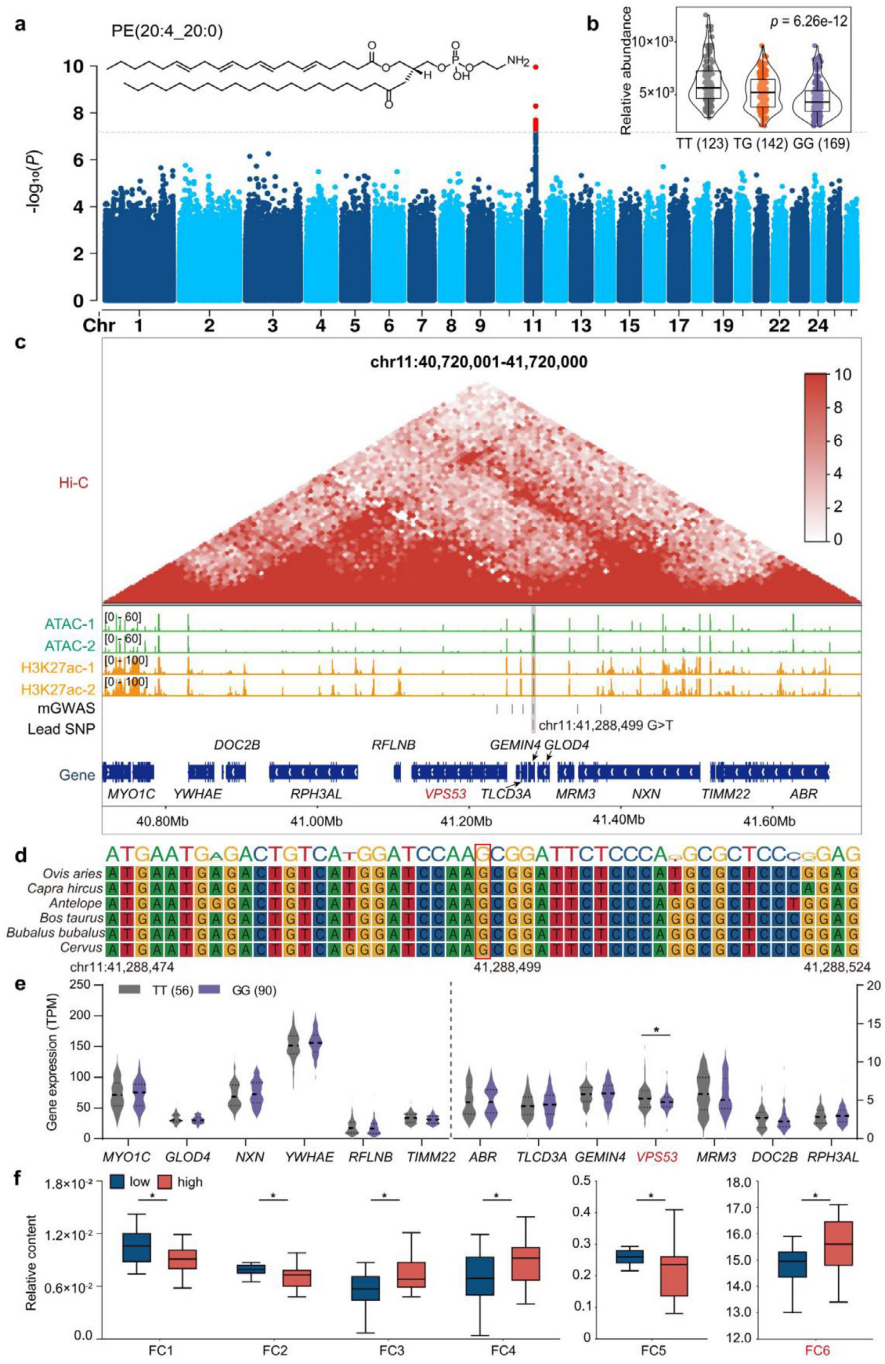
The genetic regulation of the levels of PE(20:4_20:0). a) Structure and mGWAS Manhattan plot of PE(20:4_20:0). The horizontal grey dashed line indicates the genome‐wide significance (*P* < 6.26 × 10^−8^) threshold. b) Comparison of lipid abundance of different genotypes of the lead SNP. The numbers of individuals are given in brackets. c) Integrative analysis of multi‐omics data showing the Hi‐C interaction heatmap of Chr11: 40720001–41720000 region at 10 Kb resolution, and the epigenetic signal of ATAC‐seq, H3K27ac in muscle, two replicates for each assay. The values in brackets (left) indicate ATAC‐seq and H3K27ac signals intensities. d) Conservation analysis of the lead SNP for PE(20:4_20:0) and the flanking sequences across ruminant species. e) Comparison of gene expression levels within the TAD region. The numbers of individuals are given in brackets. f) Relative content of differentially volatile compounds. FC1: 6‐methyl‐2,4‐heptanedione, FC2: 6‐methyl‐5‐hepten‐2‐one, FC3: 4(1H)‐pyridone, FC4: geranic acid, FC5: (*Z*)‐8‐methyl‐2‐decene, FC6: dihydro‐2‐methyl‐3(2H)‐furanone. *P < 0.05.

PEs, which generally comprise long‐chain polyunsaturated fatty acids, have been identified as the key lipids affecting meat flavor quality.^[^ ^26^, ^27^ ^]^ According to mGWAS, a QTL associated with the level of a PE containing eicosatetraenoic acid (PE: 20:4_20:0) was mapped to Chr11: 41.24–41.38 Mbp (**Figure** **6**a; Figure S20a,b, Supporting Information). The lead SNP (Chr11: 41288,499 bp) with the highest association with PE(20:4_20:0) levels explained 9.20% of the total variance (Figure 6b). Pairwise LD analysis of the SNPs within this QTL region revealed that only one SNP, located ≈4.1 kb downstream of the lead SNP, exhibits a strong linkage (pairwise *r^2^* > 0.6) (Figure S20c, Supporting Information). To identify potential candidate genes and causal variants, we integrated mGWAS results with epigenomic profiles (ATAC‐seq, H3K27ac, and Hi‐C) and transcriptome data from sheep LT muscle. The results demonstrated that the lead SNP, which is highly conserved across ruminants, was localized within active enhancer elements (marked by ATAC‐seq and H3K27ac signals) (Figure 6c,d). Furthermore, this SNP co‐localized within the same topologically associating domain (TAD) as 13 genes (Figure 6c). Comparative analysis of gene expression levels in this TAD revealed that only *VPS53* exhibited a significant difference between homozygous carriers for the lead SNP (Figure 6e). Notably, *VPS53* has been reported to be involved in the uptake of PE in eukaryotic plasma membranes by forming the Golgi‐associated retrograde protein (GARP) complex.^[^ ^28^ ^]^ These results suggest that the lead SNP may alter enhancer activity, modulating *VPS53* expression through chromatin interactions, thereby influencing the PE levels in sheep muscle.

GC‐MS analysis of samples from the groups with an extreme abundance of PE(20:4_20:0) revealed that six volatile compounds exhibited significant differences in content (Figure 6f; Table S12, Supporting Information). Among these, dihydro‐2‐methyl‐3(2H)‐furanone (FC6) had rOAV values > 1, with higher values observed in the high (3.14 × 10^6^) compared to the low (2.95 × 10^6^) lipid abundance group. This suggests that elevated PE(20:4_20:0) levels may enhance sweet, bread‐like, buttery, and nutty flavors in sheep meat.

## 3 Discussion

Meat flavor is a complex trait that arises from the interaction of various compounds with distinct aromas, and lipids play indispensable and prominent roles in the formation of flavor compounds.^[^ ^29^, ^30^, ^31^ ^]^ Identifying the genetic variants controlling lipids and their relationship with meat flavor is one of the many challenges in ruminant livestock. Here, we constructed the first comprehensive genome‐wide genetic atlas of the muscle lipidome through systematic profiling of 434 sheep. Strikingly, substantial inter‐individual variation in lipid profiles was observed, suggesting limited historical selection intensity on muscle lipids, alongside the discovery of high heritability for most lipids that confer significant potential for flavor improvement through selection. Building upon these findings, we identified a series of significant QTLs and candidate genes that can provide foundational insights into the genetic architecture of meat flavor traits, with implications for enhancing the economic value and culinary appeal of ruminant meat.

Lipid profile variation fundamentally determines flavor differences in lamb meat.^[^ ^27^ ^]^ This study establishes the first systematic characterization of muscle lipid profiles in ruminants across a large phenotyped population, providing crucial insights into understanding meat flavor variations. The extreme phenotype model using GP/GL stratification was constructed to assess the impacts of major lipid on meat flavor, demonstrating that GP exerts a more pronounced influence. This effect may be mechanistically linked to their oxidation‐prone polyunsaturated acyl chains, which generate flavor compounds through peroxidation.^[^ ^32^ ^]^ Notably, ruminants’ higher muscle GP content compared to non‐ruminants^[^ ^22^ ^]^ may underlie their distinctive flavor complexity. We observed distinct lipid‐flavor interactions: GL enhances ketones and aldehydes, whereas GP reduces these compounds but increases alcohols. This divergence likely stems from GL's hydrophobic nature enabling stable oxidation pathways, contrasting with GP's polar groups that increase oxidative susceptibility.^[^ ^33^ ^]^ Crucially, as membrane components, GP maintains homeostasis; their extreme fluctuations alter membrane protein levels, cellular physiology characteristics, and energy metabolism, thereby impacting water‐soluble flavor precursors (such as amino acids and carbohydrates).^[^ ^34^ ^]^ Subsequent Maillard reaction intermediates from these precursors further suppress lipid oxidation, ultimately diminishing flavor compound levels.^[^ ^29^ ^]^ This study marks the first exploration of the realistic impact of variations in major lipids on overall meat flavor characteristics within a large‐scale dataset, taking into account the complex physiological environment and interactions among various metabolites.

Lipid profiles in animals are relatively stable due to lipid homeostasis. Consequently, the heritability of certain lipids can be quite high, offering opportunities to identify the genetic factors driving their variation.^[^ ^35^ ^]^ Our subsequent baseline investigation revealed that over half of the muscle lipids had moderate to high heritability (*h^2^* > 0.2), with 12.57% showing strong genetic influence (*h^2^* > 0.5). These highly heritable lipids (predominantly GPs) generally occupy core regulatory nodes in metabolic networks, participating in diverse physiological processes where their homeostasis is critical for survival, thus likely being under stronger genetic constraint. This substantial heritability provides a foundation for improving meat flavor traits through targeted modulation of lipid metabolism. We also observed significant heterogeneity in lipid category heritability, with higher *h^2^* for GPs compared to that of GLs, SPs, and FAs, as previously reported.^[^ ^36^ ^]^ This finding aligns well with previous research that found significantly higher *h^2^* estimates in lipids containing polyunsaturated fatty acids, driven by a more complex acyl structure.^[^ ^36^, ^37^ ^]^ Notably, heritability estimates for some lipids converged at the boundary (*h^2^* approaching 0), potentially due to limited sample size. While lipids’ closer genotype‐phenotype proximity typically requires smaller cohorts (usually a few hundred) than complex trait GWAS,^[^ ^38^ ^]^ previous reports on complex traits like meat quality demonstrate that boundary convergence resolves with larger datasets.^[^ ^39^, ^40^ ^]^ Expanding sample sizes will improve genetic parameter precision in future studies.

Through mGWAS, we identified 467 genetic variants associated with 233 lipids and uncovered several previously reported and novel regulatory genes. While prior studies suggest that genetic variations in primary metabolites are typically regulated by numerous small‐effect genes,^[^ ^41^, ^42^ ^]^ our analysis revealed a key finding that a substantial proportion of lipid levels (particularly in highly correlated lipid clusters) are predominantly governed by master metabolic regulator genes (Figure 2a). A typical example includes 13 TGs and one DG governed by pleiotropic loci linked to *SH2D4A*, which has previously been implicated in human blood TG metabolism.^[^ ^43^ ^]^ *SH2D4A* modulates *STAT3* signaling, thereby driving TG and DG synthesis (Figure 5h).^[^ ^44^ ^]^ These major lipid components of IMF displayed strong positive genetic correlations with IMF content, where increased IMF accumulation paradoxically reduces aroma release due to flavor compounds’ lipophilicity,^[^ ^25^, ^45^ ^]^ which aligns with the reported inverse TG/DG‐flavor relationships in sheep.^[^ ^22^ ^]^ Notably, these master genes exhibited cross‐hierarchical pleiotropy, simultaneously modulating downstream meat quality traits; all 13 TGs displayed positive genetic correlations with meat color parameters (L^*^_24 h_ and b^*^_24 h_), while 12 TGs showed negative correlations with pH_45min_ (Figure S19, Supporting Information). This multilayered regulation suggests that major‐effect genes act as central regulators, controlling key lipid metabolism steps that ultimately influence phenotypic traits.

Contrasting the aforementioned results, indicating that the master regulator exhibited global effects within the same lipid class, some equally pleiotropic genes appear to influence specific types of lipids. In this study, *MBOAT1* and *PAQR8* demonstrated acyl chain‐specific regulation of MGDGs, which can be attributed to their functional specificity. As a C18:1‐preferring LPS acyltransferase,^[^ ^18^ ^]^ *MBOAT1* catalyzes LPS(18:1_0:0) acylation to phosphatidylserine(C18:1). Consistent with this role, we identified a coordinated expression pattern where *MBOAT1*, phosphatidylserine decarboxylase (*PISD*), and alpha‐galactosidase (*GLA*) collectively regulate C18:1‐MGDGs, with individuals carrying the G1 genotype exhibiting significantly higher expression of these three genes compared to G4 individuals (Figure S21a, Supporting Information). This synergistic upregulation facilitates PS(C18:1) flux through *PISD* to DG(C18:1), followed by *GLA*‐mediated conversion to MGDG(C18:1) (Figure 3h). Further supporting this pathway, the phosphatidic acid phosphatase 3 (*PLPP3*), responsible for DG generation, also shows a specific significant positive correlation with MGDG(C18:1) levels (Figure S21c, Supporting Information).^[^ ^46^ ^]^ *PAQR8* regulates MGDG biosynthesis through dual pathways (Figure 4h). As an adiponectin receptor, its reduced expression inhibits *AdipoQ*‐mediated fatty acid β‐oxidation,^[^ ^47^ ^]^ increasing fatty acid availability for MGDG synthesis. Second, as a ceramidase, its downregulation inhibits ceramide hydrolysis,^[^ ^20^, ^47^ ^]^ elevating ceramide levels. Notably, individuals with the *PAQR8*‐linked AA genotype (Chr20: 26333319) show significantly reduced *PAQR8* expression but elevated sphingomyelin synthase (*SGMS1*) expression versus GG individuals (Figure S21b, Supporting Information). This inverse relationship promotes ceramide conversion to DG via *SGMS1*,^[^ ^48^ ^]^ ultimately forming MGDG(C18:2). Critically, *PLPP4* specifically provides C18:2‐DG substrates for this pathway, evidenced by its exclusive significant positive correlation with MGDG(C18:2) (Figure S21d, Supporting Information). Overall, these variants selectively modulate key biosynthetic enzymes within discrete pathways, enabling precise acyl‐chain regulation and providing important clues for the target regulation of flavor‐related lipids. However, the mechanisms by which SNP variation regulates gene expression are highly complex. Although we employed multiple epigenomic approaches (including ATAC‐seq, CUT&Tag, and Hi‐C) combined with experimental validation to investigate how candidate SNPs regulate the *PAQR8* gene (Figure S13b,c, Supporting Information), our findings remained inconclusive. This uncertainty might be attributable, at least in part, to limitations in the epigenetic modification markers analyzed. Nevertheless, our results still provide valuable insights for future mechanistic studies at this locus.

MGDGs are minor components relative to glycosphingolipids and can be inadvertently destroyed during some isolation procedures for the analysis of the latter, which often leads to them being overlooked in studies of animal glycolipids.^[^ ^49^ ^]^ This study is the first to find that variations in MGDGs significantly impact ruminant meat flavor. However, since flavor formation involves complex biochemical reactions, the chemical roadmap among these lipids and flavor compounds remains uncertain, which is a limitation of the current study. Although GPs have a greater influence on meat flavor, we did not identify stronger causative variants, and this may require future and further investigations in larger test populations. The results in this study may however be of broader relevance and serve as a valuable reference for studies in other ruminant species, for the following reasons: (1) ruminant meat lipid profiles are generally similar across species;^[^ ^50^ ^]^ (2) the key pathways of lipid metabolism are evolutionarily conserved in mammals; and (3) of more relevance, ruminant genomes demonstrate substantial conservation.^[^ ^51^, ^52^ ^]^ Specifically, the peak SNPs identified in this study and their flanking sequences are conserved among multiple ruminant species (Figure 6d; Figures S8, S16, Supporting Information).

## 4 Conclusion

In conclusion, our results present a comprehensive lipidomics and volatilomics analysis of sheep meat, enhancing our understanding of the genetic basis of meat flavor traits. We have created a valuable roadmap linking lipids to genetic variations. This detailed catalog of lipid associations offers novel opportunities to study the role of flavor‐associated loci with lipids and contributes to the development of precision breeding strategies for enhancing ruminant meat flavor and quality.

## 5 Experimental Section

### Animal Management and Sample Collection

A total of 434 male Hu sheep reared on the same farm were used in this study. After weaning at two months, all sheep were transferred to the Minqin experimental farm of Lanzhou University (N38°43′41″, E103°013′), where they were raised under the same management and fed ad libitum by total mixed ration pellets.^[^ ^53^ ^]^ At the age of 180 days, all the animals with an average live weight of 48.1 ± 6.36 kg were slaughtered after 16 h of fasting, but with free access to water in accordance with the agricultural industry standard of the People's Republic of China (NY/T3469‐2019). *Longissimus thoracis* (LT) tissue samples were collected from the left side of each carcass immediately after slaughter and stored at −80 °C. Whole blood samples were also collected via jugular venipuncture and stored at −20 °C until DNA extraction. Detailed sample information is provided in Table S13 (Supporting Information).

### Lipidomics Analysis

Mass spectrometry‐based lipid analysis was performed for 434 samples by a widely targeted lipidomics approach at Metware Biotechnology Co., Ltd. (Wuhan, China; https://www.metwarebio.com/). The lipids were extracted following Liu et al.^[^ ^15^ ^]^ In brief, the LT samples were thawed on ice and pulverized in liquid nitrogen, and 20 mg of the resulting powder was then mixed with 1 mL of extraction solvent (MTBE:MeOH = 3:1, v/v) containing isotope‐labeled internal standards, followed by vortex mixing for 15 min. Then, 200 µL of water was added, and the mixture was vortexed for 1 min. The sample was then centrifuged at 12,000 rpm for 10 min, the supernatant (200 µL) was collected and vacuum‐dried. The dry extract was reconstituted using 200 µL mobile phase B (acetonitrile/isopropanol, 10/90 V/V, with 0.1% formic acid and 10 mmol/L ammonium formate) prior to LC‐MS/MS analysis.

Lipids were analyzed using an LC–ESI–MS/MS system (UPLC, ExionLC AD; MS, QTRAP System) with a Thermo Accucore C30 (2.6 µm, 2.1 mm × 100 mm) column. To monitor and evaluate stability and reliability during lipid detection, quality control (QC) samples were prepared by mixing all 434 samples and inserted into the detection queue. The experimental conditions were as follows: the solvent system consisted of A (acetonitrile/water, 60/40, V/V, with 0.1% formic acid and 10 mmol L^−1^ ammonium formate) and B (acetonitrile/isopropanol, 10/90 V/V, with 0.1% formic acid and 10 mmol L^−1^ ammonium formate) buffers; the gradient program started with A/B (80:20, V/V) at 0 min, followed by 70:30 V/V at 2.0 min, 40:60 V/V at 4 min, 15:85 V/V at 9 min, 10:90 V/V at 14 min, 5:95 V/V at 15.5 min, 5:95 V/V at 17.3 min, 80:20 V/V at 17.5 min, and finally 80:20 V/V at 20 min. The flow rate was set at 0.35 mL/min; the temperature was maintained at 45 °C; and the injection volume was 2 µL. The eluent was passed through an ESI‐triple quadrupole‐linear ion trap (QTRAP) ‐MS system, featuring an electrospray ionization (ESI) Turbo Ion‐Spray interface. The system operated in both positive and negative ion modes and was controlled by Analyst 1.6.3 software (AB Sciex, Framingham, US). Instrument tuning and mass calibration were performed with 10 and 100 µmol/L polypropylene glycol solutions in QQQ and LIT modes, respectively. QQQ scans were acquired as multiple reaction monitoring (MRM) experiments with collision gas (nitrogen) set to 5 psi. The declustering potential (DP) and collision energy (CE) for individual MRM transitions were determined with further DP and CE optimization. A specific set of MRM transitions was monitored in each period based on the elution of lipids during that time frame. Specific ion pairs were established for each lipid, and the retention time (RT) was determined. Under an extended gradient separation, each lipid eluted at its dedicated RT. Lipids were identified using the Metware database (MWDB), the proprietary standard substance database of Metware Biotechnology Co., Ltd., utilizing RT, precursor ion pair information, and secondary spectral data.^[^ ^54^ ^]^ The relative contents of each lipid were represented as normalized peak areas.

### Gas Chromatography‐Ion Mobility Spectrometry (GC‐IMS) Analysis

GC‐IMS, ideal for exploratory comparisons and pattern recognition due to its strength in real‐time volatile compound fingerprinting without requiring prior identification,^[^ ^55^ ^]^ was employed to rapidly differentiate global flavor profiles between high‐ and low‐ GL/GP groups. Forty LT samples were randomly selected from the top and bottom five percentiles of GL and GP contents respectively, then systematically divided into four experimental groups (10 samples per group) – GL_H (highest GL content), GL_L (lowest GL content), GP_H (highest GP content), and GP_L (lowest GP content) – for comparative analysis of volatile compounds using a FlavourSpec GC‐IMS system (G.A.S. Instrument, Dortmund, Germany). The samples were thawed at 4 °C overnight before the experiments. The measurement procedure followed the protocol outlined by Wang et al.,^[^ ^55^ ^]^ with slight modifications. Samples weighing 2.0 g were placed into a 20 mL headspace vial with a magnetic screw seal cover. The samples were then incubated at 80 °C for 20 min. Volatile compounds were separated on a 15 m × 0.53 mm column (FS‐SE‐54‐CB‐1, CS‐Chromatographie Service GmbH, Langerwehe, Germany) maintained at 60 °C. Nitrogen carrier gas (99.999% purity) was used with the programmed flow: 2 mL min^−1^ (2 min), 15 mL min^−1^ (8 min), 100 mL min^−1^ (10 min), and 150 mL/min (5 min). The identification of volatile compounds involved comparing the retention index (RI; calculated with n‐ketones C4‐C9 as an external standard) and the drift time (Dt; the time required for ions to reach the collector through the drift tube) of the standard in the GC‐IMS library.^[^ ^56^ ^]^ The fingerprint map of the analytical spectrum was acquired and processed on a laboratory analytical viewer (LAV, G.A.S., Dortmund, Germany). Each test was done thrice to ensure the accuracy and reliability of the results.

### Whole‐Genome Sequencing and Genotyping

Genomic DNA was extracted from blood samples using a standard phenol‐chloroform method.^[^ ^57^ ^]^ After quality control, 434 qualified DNA samples were subjected to whole‐genome resequencing using the Illumina HiSeq X Ten platform (PE150) at Novogene Corporation (Beijing, China). After resequencing, low‐quality reads were removed by Trimmomatic version 0.36,^[^ ^58^ ^]^ and clean reads were aligned to the sheep reference genome (*Oar_rambouillet_v1.0*) with Burrows‐Wheeler‐Alignment Tool (BWA, version 0.7.15)^[^ ^59^ ^]^ using default parameters. Duplicate reads were marked and removed using SAMBAMBA (https://github.com/lomereiter/sambamba) and indexed using SAMtools (http://github.com/samtools/samtools). After mapping, SNP calling was performed using the Genome Analysis Toolkit (GATK, version 3.8)^[^ ^60^ ^]^ HaplotypeCaller and GenotypeGVCFs module, and the output was further quality checked with the VariantFiltration module with the following criteria: QualByDepth (QD) > 10.0, FisherStrand (FS) < 60.0, RMS Mapping Quality (MQ) > 40.0, Mapping Quality Rank Sum Test (MQRankSum) > −12.5, Read Pos Rank Sum Test (ReadPosRankSum) > −8.0. Subsequently, the SNP dataset was filtered based on the following criteria: (1) Minor allele frequency > 0.05, (2) the maximum missing rate was < 0.2, and (3) SNPs were biallelic. A total of 10010427 high‐quality SNPs across 26 autosomes of 434 sheep were prepared for subsequent analysis. SNP annotation was performed using snpEff software (version 4.2),^[^ ^61^ ^]^ categorizing SNPs into introns, exons, intergenic regions, and upstream or downstream regions. Exonic SNPs were further categorized as synonymous or non‐synonymous.^[^ ^62^ ^]^

### Coefficient of Variation and Genetic Parameter Estimation of Lipids

The coefficient of variation (CV) was calculated for each lipid using the formulae: s/m, where “s” and “m” represent the standard deviation and mean of each lipid in all sheep, respectively. The heritability (*h^2^*) of each lipid was estimated by fitting a linear mixed animal model to the data using restricted maximum likelihood (REML) methods with ASReml‐R version 4.^[^ ^63^ ^]^ The formula applied in the analysis was as follows: *h^2^* = var(G)/var(G)+var(E), where var(G) and var(E) are the variances derived from genetic and environmental effects, respectively. The kinship matrix (matrix H) was constructed based on both pedigree (matrix A) and genome information (matrix G).^[^ ^64^ ^]^ Bivariate animal models were employed to estimate covariance components and the genetic and phenotypic correlations among co‐localized lipids. A likelihood ratio test (LRT) was used to determine whether the correlations of the given phenotype were significant (*P_LRT_* < 0.05).

### Metabolome‐based genome‐wide association (mGWAS) and linkage disequilibrium (LD)

The mGWAS was performed using a mixed linear model in rMVP (The Memory‐efficient, Visualization‐enhanced, and Parallel‐accelerated tool) R package.^[^ ^65^ ^]^ To approximate a normal distribution, the relative content of lipids was log_2_‐transformed as phenotypic values.^[^ ^15^ ^]^ In rMVP, the population structure was characterized using the first three PCs (Q matrix) to incorporate this information with the VanRaden kinship matrix as fixed and random effects, respectively. The effective number of independent SNPs was calculated using PLINK with parameters (–indep‐pairwise 50 5 0.2), and the genome‐wide significant and suggestive thresholds were set to 6.26 × 10^−8^ (0.05/798133) and 1.25 × 10^−6^ (1/798133), respectively.^[^ ^66^ ^]^ The proportion of variance explained (PVE) by the identified SNP loci was calculated using the formula:^[^ ^67^ ^]^

(1) $$PVE=\frac{2{\hat{\beta }}^{2}MAF\left(1-MAF\right)}{2{\hat{\beta }}^{2}MAF\left(1-MAF\right)+{\left(\mathrm{se}\left(\hat{\beta }\right)\right)}^{2}2NMAF\left(1-MAF\right)}$$

where, $\hat{\beta }$ is the effect size, *MAF* is the minor allele frequency, $\mathrm{se}(\hat{\beta })$ is the standard error of effect size, and *N* is the sample size. LD coefficients based on *r^2^* values between the SNPs within 1 Mbp of the lead SNP were calculated by PLINK (version 1.90) software.^[^ ^68^ ^]^

### RNA‐sequencing and candidate gene identification

LT muscles from 210 sheep, selected at random from the 434 sheep, were used for transcriptomic sequencing. Total RNA was extracted using TRIzol RNA Reagent (Takara, Dalian, China), and its concentration and integrity were assessed using the Agilent 2100 RNA 6000 Nano Kit (Agilent Technologies, Waldbronn, Germany). Qualified RNA samples were reverse transcribed into cDNA to construct libraries, which were subsequently sequenced on the Illumina X‐Ten platform, producing 150‐bp paired‐end reads. To ensure the quality of sequences, raw reads containing low‐quality bases or adaptor contamination were eliminated using Trimmomatic software (version 0.36), and the Q20 and Q30 values were calculated to evaluate the quality of the clean data. The filtered sequences were aligned to the *Ovis aries* reference genome (*Oar_rambouillet_v1.0*) using HISAT2 software (version 2.0.1).^[^ ^69^ ^]^ The transcripts per kilobase of the exon model per million mapped reads (TPM) for each gene were calculated using the featureCounts package.^[^ ^70^, ^71^ ^]^

Candidate genes were identified through a tripartite screening strategy: (1) differential expression analysis of genes within the 1 Mbp flanking region of the lead SNP across genotypic groups,^[^ ^72^ ^]^ (2) significant correlation between gene expression level and lipid abundance, and (3) prior knowledge.

### Hi‐C Library Construction, Sequencing, And data Analysis

LT tissues from two Hu sheep were randomly selected to perform the in situ Hi‐C experiment according to Zhao et al., with minor modifications.^[^ ^73^ ^]^ ≈1 g of the sample was pulverized in liquid nitrogen and fixed with 1% formaldehyde (Sigma‐Aldrich, 252 549) at room temperature for 20 min, and then quenched with 0.125 M glycine (Sigma, V900144). The crosslinked samples were lysed in ice‐cold Hi‐C buffer (10 mM Tris‐HCl pH 8.0 [Invitrogen, 15 568 025], 10 mM NaCl [Invitrogen, AM9759], 0.2% NP‐40 [Thermo Scientific, 85 124]) for 15 min, followed by treatment with 0.3% SDS at 62 °C for 5–10 min, and the reaction was halted with Triton X‐100 (Thermo Scientific, 28 314). Chromatin was digested with AluI endonuclease (NEB, R0137L) at 37 °C for 7 h, followed by labeling of the DNA fragment ends with biotin (0.4 mm biotin‐14‐dATP [Life Technologies, 19524‐016], 10 mM dCTP, 10 mM dGTP, 10 mM dTTP, 5 U µL^−1^ DNA Polymerase I [NEB, M0210]) by incubation at 37 °C for 1 h. Ligation was performed with T4 DNA ligase (NEB, M0202L) at 16 °C overnight. Biotinylated DNA was sheared to 300–500 bp using a Covaris S220, and libraries were prepared following Illumina protocols (Illumina, San Diego, CA) and sequenced on a NovaSeq 6000 platform.

The paired‐end Hi‐C reads from different libraries were aligned and filtered with the HiC‐Pro (version 2.11.4) pipeline.^[^ ^74^ ^]^ We then merged the multiple libraries, built raw inter‐/intra‐chromosomal contact maps, and performed matrix balancing iterative correction and eigenvector decomposition (ICE) normalization. Using HiC‐Pro, we constructed a 40‐kb resolution interaction matrix for individual chromosomes and performed normalization. The Insulation Score (IS) was calculated using Cworld‐dekker software (version 0.41.1) to identify TAD boundaries for each chromosome.^[^ ^75^ ^]^ Regions between two adjacent TAD boundaries were defined as individual TADs. Genome‐wide loop structures were detected using HiCCUPS (version 0.3.4) by analyzing pairwise interactions between bins.^[^ ^73^ ^]^ Significant interaction sites were defined as loop anchors separated by <2 Mb.

### ATAC‐seq and CUT&Tag‐seq

The ATAC‐seq and CUT&Tag‐seq datasets used in this study originate from Zhang et al.^[^ ^52^ ^]^ For ATAC‐seq, ≈5 mg of LT tissue was pulverized in liquid nitrogen. The powder was suspended in 1 mL ice‐cold PBS and centrifuged. Nuclei were extracted, incubated with Tn5 transposase reaction mix at 37 °C for 1 h, and purified using a DNA Purification and Concentration Kit (TD413; Genstone Biotech). The transposed DNA fragments were then amplified by PCR with NEBNext High‐Fidelity 2X PCR Master Mix (M0541L; NEB), purified using Kapa Pure Beads (KS8002; Kapa Biosystems), and sequenced on an Illumina NovaSeq 6000 platform (Wuhan Yingzi Gene Technology).

For CUT&Tag‐seq, nuclei extracted from flash‐frozen tissues (using the ATAC‐seq method) were mixed with concanavalin A‐coated magnetic beads (BP531; BioMag Plus), incubating at room temperature for 20 min. Nuclei‐bound beads were incubated with H3K27ac antibodies (ab4729; Abcam) at room temperature for 1 h. After washing, samples were incubated with secondary antibody‐conjugated beads (goat anti‐rabbit IgG, ab6702; Abcam) at room temperature for 1 h. Subsequently, Protein G‐Tn5 transposase complex (Hyperactive pG‐Tn5 Transposase; S602, Vazyme) was added and incubated at room temperature for 1 h. Following washing, tagmentation was activated in Mg^2+^‐containing buffer (AM9530G; Invitrogen) at 37 °C for 1 h. The reaction was stopped with SDS Buffer (15553–027; Invitrogen). DNA was extracted using Tagment DNA Extract Beads (N245; Novoprotein), amplified by PCR (NEBNext High‐Fidelity 2X PCR Master Mix; M0541L, NEB), purified with Kapa Pure Beads (KS8002; Kapa Biosystems), and sequenced on an Illumina NovaSeq 6000 platform (Wuhan Yingzi Gene Technology).

After quality control, peaks were called for each replicate individually. Significant peaks (*P* < 0.00001) were retained for further analysis and annotated against the sheep reference genome (*Oar_rambouillet_v1.0*).

### Dual‐Luciferase Reporter Assay

Enhancer activity validation was performed using the reporter gene vector pGL4.23, which contains a minP promoter. Validated enhancer sequences (containing the key SNP with 500 bp upstream and downstream flanking regions) were cloned into the upstream region of the promoter, and the enhancer activity was determined by measuring reporter gene activity. Luciferase activity was detected 48 h post‐transfection in both 293T cells and sheep intramuscular preadipocytes. All experiments were conducted in triplicate, and the firefly luciferase activity was normalized to the Renilla luciferase activity of each sample.

### Gas Chromatography‐Mass Spectrometry (GC‐MS) Analysis

GC‐MS, leveraging its high sensitivity and structural specificity for precise identification and quantification of specific flavor‐active compounds,^[^ ^76^ ^]^ was applied to targeted analyses within extreme lipid‐abundance subgroups identified through mGWAS. For each significant genetic signal discussed in this study, individuals with extreme levels of the corresponding lipids (i.e., low and high 5%; n = 20 in each group) were selected from the 434 Hu sheep, resulting in a total of 140 LT samples (with 20 samples appearing in multiple groups) being subjected to flavor profile detection. Volatile compounds were extracted using a headspace solid‐phase microextraction (HS‐SPME) procedure. Prior to the assay, the muscle samples were thawed at 4 °C and then ground in liquid nitrogen to ensure uniform mixing. Given the sensitivity of the SPME Arrow (Agilent J&W Scientific, Folsom, CA, USA),^[^ ^77^ ^]^ 0.2 g of each sample was weighed into a headspace bottle, followed by the addition of 0.2 g of NaCl powder and 20 µL (10 µg mL^−1^) internal standard solution (3‐Hexanone‐2,2,4,4‐d4). The samples were then incubated at 80 °C for 5 min, and a 120 µm DVB/CWR/PDMS extraction head was inserted into the headspace bottle for 15 min for headspace extraction. Subsequently, the sample was desorbed at 250 °C for 5 min and then separated and identified using the Agilent Model 8890 GC and a 7000D mass spectrometer. Prior to sampling, the DB‐5MS capillary column (30 m × 0.25 mm × 0.25 µm, Agilent J&W Scientific, Folsom, CA, USA) was aged in the fiber conditioning station at 250 °C for 5 min. Helium was used as the carrier gas at a linear velocity of 1.2 mL min^−1^. The injection port temperature was set at 250 °C, and a solvent delay of 3.5 min was applied. Temperature programming was as follows: 40 °C for 3.5 min, 10 °C min^−1^ to 100 °C, 7 °C min^−1^ to 180 °C, and finally 25 °C min^−1^ to 280 °C for 5 min. MS was performed using electron impact ionization (EI) at 70 eV. The scan range was from 30 to 400 m z^−1^ with an automatic‐gain‐control target value of 1E6. Ion source temperature and spectrometry interface temperature were set at 230 and 280 °C, respectively. To ensure instrument stability, a master mix QC sample, prepared by mixing an equal quantity of each test sample, was added for every 10 samples tested. The original data after mass spectrometry analysis were processed for qualitative and quantitative analysis by MassHunter software (B.08.00). Volatile compounds were identified in accordance with mass spectra and linear retention indices from NIST17 version 2.3, Wiley9, and a domestic library built with authentic reference standards. Flavor attributes were annotated using consensus descriptors from published literature^[^ ^8^, ^78^, ^79^ ^]^ and authoritative flavor chemistry databases (Perflavory Database, Leffingwell Odour Database, Food Flavor Laboratory, and The Good Scents Company). Aroma compounds were semi‐quantified using internal standards, with content expressed in µg/g. The rOAV for each compound was subsequently calculated based on the semi‐quantitative data.

### Statistical analysis

Data were presented as the mean ± standard error. Significant differences in volatile compound content and dual‐luciferase activity were determined using Student's t‐tests, while differences in lipid abundance and gene expression among the genotypes were compared by one‐way ANOVA followed by Tukey's post hoc test in SPSS 22.0 software (SPSS, Inc., Chicago, IL, USA). Statistical significance was denoted by the following symbols: ^*^ *P* < 0.05, ^**^ *P* < 0.01, ^***^ *P* < 0.001. Spearman's correlation between lipids was determined through pairwise comparison by the Hmisc package in R version 3.6.1. Using machine learning methods, partial least squares‐discriminant analysis (PLS‐DA) was employed to construct calibration models aimed at discriminating between groups with varying GL/GP content.^[^ ^80^ ^]^ This analysis was conducted using in‐house algorithms developed in the MATLAB R2020a environment (The Mathworks, Natick, MA, USA). The sensitivity and specificity values from various classes were used to evaluate the performance of the PLS‐DA models.

## Funding

This work was supported by the National Natural Science Foundation of China (32 072 713), the Gansu Youth Science and Technology Fund Program (24JRRA440), and the China Postdoctoral Science Foundation (2024M751265), the earmarked fund for China Agriculture Research System (CARS‐38) and Precision Identification Project of Livestock and Poultry Genetic Resources (202105). The Chinese Government contribution to ICARDA (through J.M.M.) is appreciated. The paper contributes to the CGIAR SAAF Science Program supported by the CGIAR Trust Fund J.M.M also acknowledges the support of SRUC (UK).

## Conflict of Interest

The authors declare no conflict of interest.

## Author contributions

X.Z., Y.K., and J.C. contributed equally to this work. X.Y. conceptualized the project. X.Z., X.Y., and F.L. designed the project. X.Z. wrote the original draft. X.Z., Y.K., J.C., F.W., and R.L. developed the methodology. X.Z., Y.K., Z.W., S.P., Y.L., and Q.F. collected the phenotype data. Z.W., Y.S., T.D., Y.F., B.J., J.Z., S.P., Y.L., and Q.F. curated the generated data. X.Z., Y.K., J.C., and H. Z. carried out data analysis and handled visualization. X.Z., R.L., W.F., M.J., and X.Y. did the writing, reviewing, and editing of the manuscript. X.Y. provided supervision, managed project administration, and secured funding.

## Ethical statement

All experimental protocols in this study were reviewed and approved by the Ethics Committee of the College of Pastoral Agriculture Science and Technology at Lanzhou University (File No: CY20181002). Animal care, maintenance, procedures, and experimentation were conducted in strict accordance with the guidelines and regulations approved by the Welfare and Ethics Committee of the Chinese Association for Laboratory Animal Sciences.

## Supporting information



Supporting Information

Supporting Information

## Data Availability

The high‐throughput sequence data generated in this study have been deposited with the NCBI Sequence Read Archive (SRA) under accession codes PRJNA1266838 for WGS data and PRJNA1273504 for Hi‐C. RNA‐Seq data have been deposited in the Genome Sequence Archive (GSA) under accession code CRA022963. The lipidomics and volatilomics datasets are provided in the Supporting Information.
