# Supplementary material for: Comprehensive Genetic Map of Muscle Lipidome Reveals Novel Insights Into Flavor Variation in Ruminant Meat
Source: Adv Sci (Weinh). 2025 Aug 11;12(40):e06900. doi: 10.1002/advs.202506900 (PMC12561217; doi:10.1002/advs.202506900)
Supplement: Supplementary file 1 — Supporting Information [file ADVS-12-e06900-s002.pdf]

**Supporting Information**

# Comprehensive Genetic Map of Muscle Lipidome Reveals Novel Insights into Flavor Variation in Ruminant Meat

*Xueying Zhang, Yuanyuan Kong, Jialei Chen, Ran Li, Zhongyu Wang, Yali Song, Tianshu Dai, Yuxin Fu, Beixiang Jiang, Jize Zeng, Shengwei Pei, Yangkai Liu, Qianjie Feng, Weiwei Fu, Zehu Yuan, Joram Mwashigadi Mwacharo, Fadi Li, Xiangpeng Yue**

X. Zhang, Y. Kong, J. Chen, Z. Wang, Y. Song, T. Dai, Y. Fu, B. Jiang, J. Zeng, S. Pei, Y. Liu, Q. Feng, W. Fu, F. Li, X. Yue
State Key Laboratory of Herbage Improvement and Grassland Agro-Ecosystems
College of Pastoral Agriculture Science and Technology, Lanzhou University
Lanzhou, Gansu 730000, China
E-mail: lexp@lzu.edu.cn

R. Li
College of Animal Science and technology, Northwest A&F University
Yangling, Shanxi 712100, China

Z. Yuan
Joint International Research Laboratory of Agriculture and Agri-Product Safety of Ministry of Education, Yangzhou University
Yangzhou, Jiangsu 225000, China

J. M. Mwacharo
Dryland Livestock Genomics
International Centre for Agricultural Research in the Dry Areas (ICARDA)
Addis Ababa, Ethiopia

J. M. Mwacharo
Animal and Veterinary Sciences, Scotland's Rural College
Roslin Institute Building
Edinburgh, Scotland

X. Zhang, Y. Kong, and J. Chen contributed equally to this work.

**Supporting Information for**

**Comprehensive Genetic Map of Muscle Lipidome Reveals Novel Insights into Flavor Variation in Ruminant Meat**

**This file includes:**

Supplementary text

Supplementary Figure S1-S21

Supplementary Table S1-S13

## 1. Supplementary text

*1. The detailed procedure for intramuscular fat (IMF) and meat quality phenotype determination*

The *longissimus thoracis* (LT) muscle was excised from the left side of each carcass approximately 10 minutes post-slaughter for determination of intramuscular fat (IMF) content and meat quality parameters. All measurements employed three replicates except shear force analysis (ten replicates). IMF content was quantified as weight percentage of wet muscle tissue using Soxhlet extraction with petroleum ether solvent. Meat color measurements ($L^*$, $a^*$, $b^*$) were taken at 45 min and 24 h post-slaughter on the cut surface after 30 minutes of blooming at ~20°C using a Minolta chromameter (Konica Minolta Sensing Inc., Osaka, Japan; 8-mm aperture, D65 illuminant, 2° observer). Corresponding pH values were determined using a calibrated pH meter (HI99163, HANNA Instruments Inc., RI, USA). Following 24-hour chilling at 4°C (90% humidity), we measured water-holding capacity (WHC, %), shear force (N), cooking loss (%), drip loss (%), $pH_{24h}$, $L^*_{24h}$, $a^*_{24h}$, and $b^*_{24h}$. To measure the WHC, meat samples were sandwiched between two layers of gauze and placed on a platform with 18 layers of qualitative filter paper above and below, and then were subjected to 35 kg for 5 min in an RH-1000 meat pressure meter (Runhu Instrument Co., Ltd., Guangzhou, China). The WHC value was obtained by the difference in weight of the samples before and after pressure. For cooking loss assessment, samples (30-40 g, triplicates) were heated in an 80°C water bath until reaching 70°C core temperature, then re-weighed after surface moisture removal. Shear force was measured using ten 1×1×4 cm strips (parallel to muscle fibers) from cooked samples with a C-LM3 tenderness meter (Beijing Brady Technology Development Co., Ltd., Beijing, China) featuring a Warner-Bratzler blade.

*2. Genetic correlation analysis between 14 co-localized glycerides and meat quality traits*

Bivariate animal models were used to estimate covariance components and the genetic and phenotypic correlations between the 14 glycerides (13 triglycerides and one diglyceride) and meat quality traits. The model applied in the analyses was as follows:

$$\begin{bmatrix} y_1 \\ y_2 \end{bmatrix} = \begin{bmatrix} Z_1 & 0 \\ 0 & Z_2 \end{bmatrix} \begin{bmatrix} a_1 \\ a_2 \end{bmatrix} + \begin{bmatrix} e_1 \\ e_2 \end{bmatrix} \quad (1)$$

Where $y_1$ and $y_2$ are the phenotypic records vector of the traits 1 and 2; $a_1$, $a_2$, $e_1$, and $e_2$ are the vectors of additive genetic and residual effects for traits 1 and 2, respectively; $Z_1$ and $Z_2$ are incidence matrices relating traits to genetic additive effects. The variance-covariance structure of animal genetic effects was:

$$G \otimes \begin{bmatrix} \sigma_{a_1}^2 & r_g \sigma_{a_1} \sigma_{a_2} \\ r_g \sigma_{a_1} \sigma_{a_2} & \sigma_{a_2}^2 \end{bmatrix} \quad (2)$$

Where $\sigma_{a_1}$ and $\sigma_{a_2}$ represent the additive genetic standard deviations of traits 1 and 2, and $r_g$ represents their genetic correlation. A likelihood ratio test (LRT) was used to test whether the correlations of the given phenotype were significant ($P_{LRT} < 0.05$).

3. *Dual-Luciferase reporter assay*

Enhancer activity validation was performed using the reporter gene vector pGL4.23, which contains a minP promoter. Validated enhancer sequences (containing the key SNP with 500 bp upstream and downstream flanking regions) were cloned into the upstream region of the promoter, and the enhancer activity was determined by measuring reporter gene activity. Luciferase activity was detected 48 h post-

transfection in both 293T cells and sheep intramuscular preadipocytes. All experiments were conducted in triplicate, and the firefly luciferase activity was normalized to the Renilla luciferase activity of each sample.

**2. List of Supplementary Figures**

**Figure S1**. Coefficients of variation (CV) and narrow-sense heritability ($h^2$) for individual lipids in the *longissimus thoracis* of Hu sheep.

**Figure S2**. Comparative analysis of lipid class abundance in extreme phenotype models.

**Figure S3**. Effects of glycerolipids (GL) and glycerophospholipids (GP) on meat flavor profiles

**Figure S4**. Numbers of mGWAS signals for each lipid.

**Figure S5**. KEGG enrichment analysis of candidate genes and their associated lipids.

**Figure S6**. EI–MS spectrum and QQ plot of LPS(18:1_0:0), MGDG(16:0_18:1), and MGDG(16:1_18:1).

**Figure S7**. Genetic parameters estimation for LPS(18:1_0:0), MGDG(16:0_18:1), and MGDG(16:1_18:1).

**Figure S8.** Conservation analysis of the lead SNPs for LPS(18:1_0:0), MGDG(16:1_18:1), and MGDG(16:0_18:1) and their flanking sequences across ruminant species.

**Figure S9**. Analysis of SNP pairwise linkage disequilibrium (LD) patterns in highly linked regions and effects of combined genotype on lipid abundance and gene expression.

**Figure S10**. Correlation between *MBOAT1* expression and lipids abundance

**Figure S11.** Relative odor activity values (rOAVs) of differential flavor compounds in high- and low-abundance cohorts of C18:1-containing MGDG and LPS lipids.

**Figure S12.** EI–MS spectrum and QQ plot of MGDG(16:0_18:2) and MGDG(16:1_18:2).

**Figure S13.** Functional analysis of the chr20:26,333,319 locus.

**Figure S14.** EI–MS spectrum of 13 triglycerides and one diglyceride.

**Figure S15.** Manhattan plot of 13 triglycerides and one diglyceride.

**Figure S16.** Conservation analysis of the SNP associated with 13 TGs and one DG and their flanking sequences across ruminant species.

**Figure S17.** Comparative analysis of 14 glycerolipid species (13 triglycerides and 1 diglyceride) between two haplotypes.

**Figure S18.** Relative odor activity values (rOAVs) of differential flavor compounds in high- and low-abundance cohorts of 14 glycerolipids.

**Figure S19.** Correlation matrix of glycerolipids abundance with meat quality traits.

**Figure S20**. Characterization of significant mGWAS signals for PE(20:4_20:0).

**Figure S21.** Key enzymes driving acyl chain-specific MGDG biosynthesis pathways.

**3. List of Supplementary Tables**

**Table S1.** Lipids identification by widely targeted lipidomics in sheep longissimus thoracis muscle.

**Table S2.** Heritability assessment of lipids.

**Table S3.** The pairwise Spearman correlations of 947 lipid levels.

**Table S4.** Volatile flavor compounds identified by GC-IMS in glyceride/glycerophospholipid groups.

**Table S5.** Discriminant effect of PLS-DA model.

**Table S6.** Summary of genome-wide significant associations identified by mGWAS.

**Table S7.** Identification of differentially volatile compounds related to LPS (18:1_0:0), MGDG (16:0_18:1), and MGDG (16:1_18:1) levels.

**Table S8.** Identification of differentially volatile compounds related to MGDG(16:0_18:2) and MGDG(16:1_18:2) levels.

**Table S9.** Genetic parameter estimates for 14 glycerides.

**Table S10.** Identification of differentially volatile compounds related to 13 TGs and DG(16:0_16:1) levels.

**Table S11.** Correlation between glycerides and meat quality.

**Table S12.** Identification of differentially volatile compounds related to PE(20:4_20:0) levels.

**Table S13.** Detailed information of sequencing samples in this study.

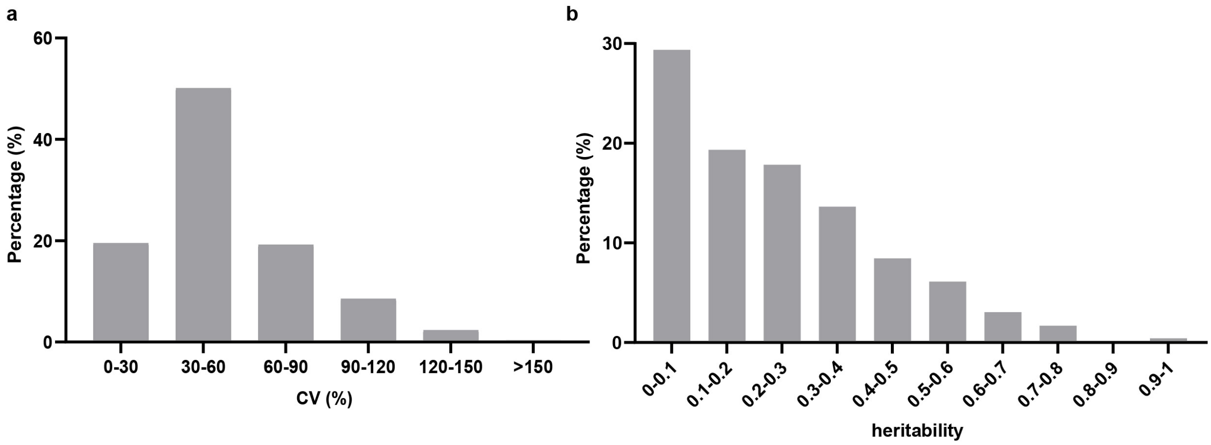


**Figure S1**. (**a**) Coefficients of variation (CV) and (**b**) narrow-sense heritability ($h^2$) for individual lipids in the *longissimus thoracis* of Hu sheep.

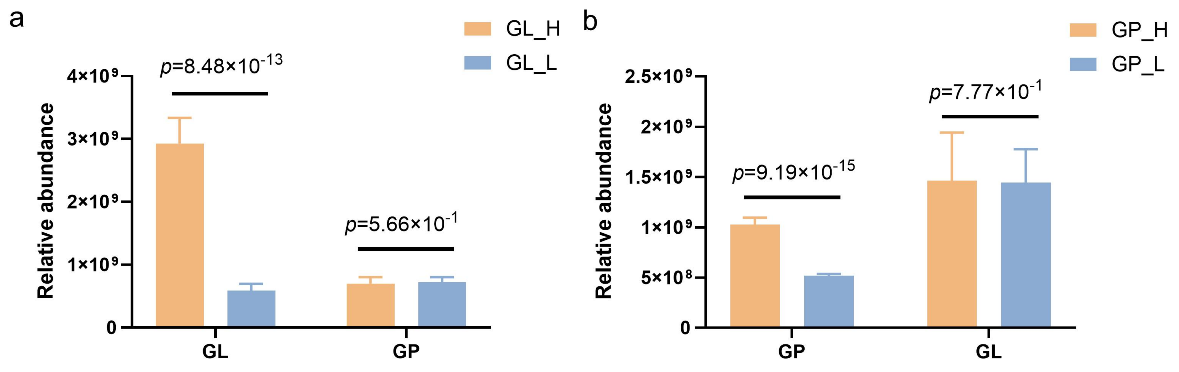


**Figure S2**. Comparative analysis of lipid class abundance in extreme phenotype models. (**a**) Glycerolipid (GL) and (**b**) glycerophospholipid (GP) abundance distributions between high- and low-abundance groups. Extreme models were defined as: GL_H (top 5% GL abundance), GL_L (bottom 5% GL), GP_H (top 5% GP), and GP_L (bottom 5% GP). Data are presented as mean ± SEM (n = 10 biological replicates per group).

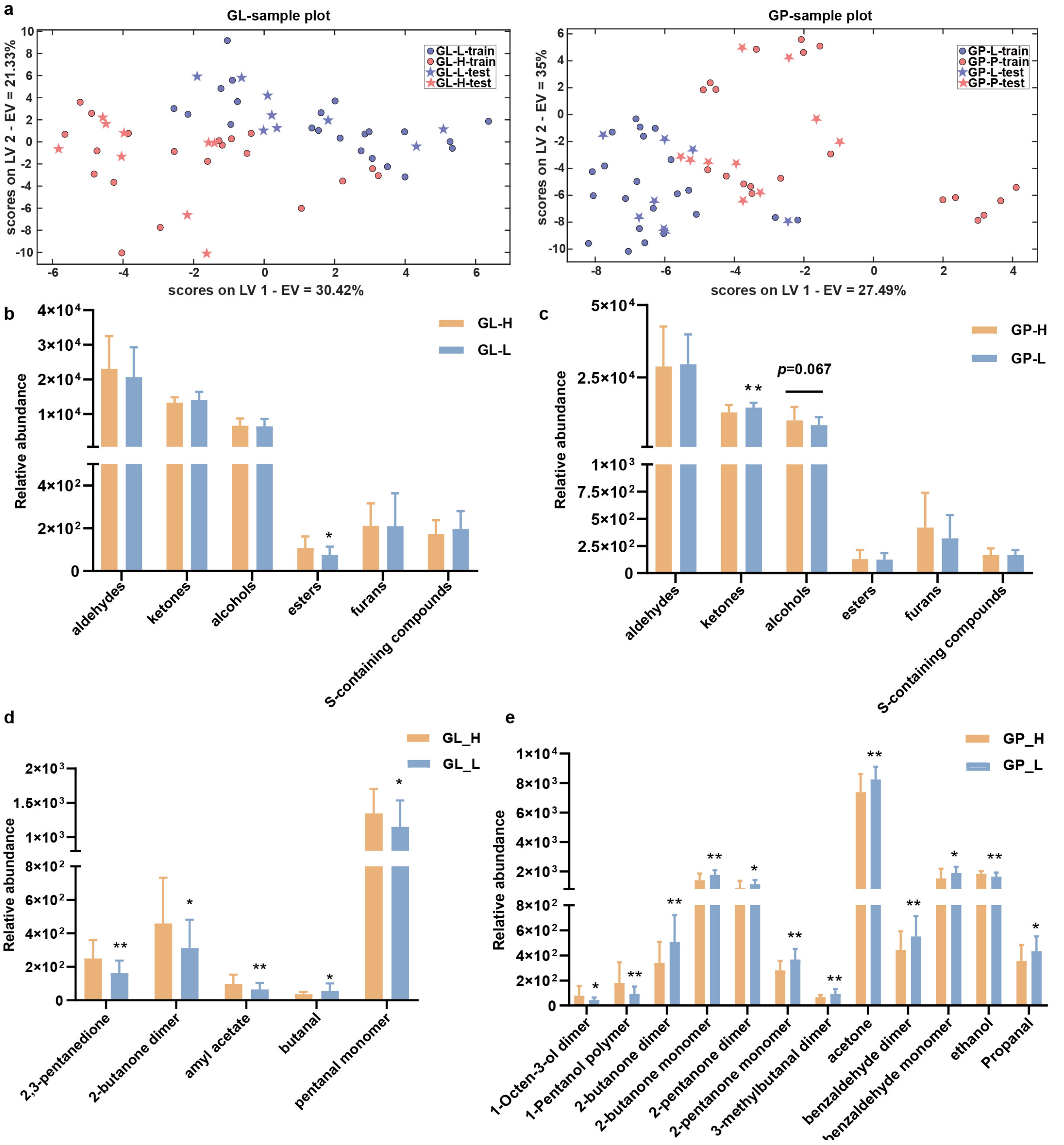


**Figure S3**. Effects of glycerolipids (GL) and glycerophospholipids (GP) on meat flavor profiles (n = 10). (**a**) Partial least squares-discriminant analysis (PLS-DA) score plot derived via machine learning algorithms. (**b-c**) Impact of GL and GP on the total volatile compound content in each class. (**d-e**) Differential abundance of volatile compounds between high/low GL/GP groups. GL_H: highest glycerolipid content; GL_L: lowest glycerolipid; GP_H: highest glycerophospholipid; GP_L: lowest glycerophospholipid. Data presented as mean ± SEM; *$P < 0.05$, **$P < 0.01$ (two-tailed t-test).

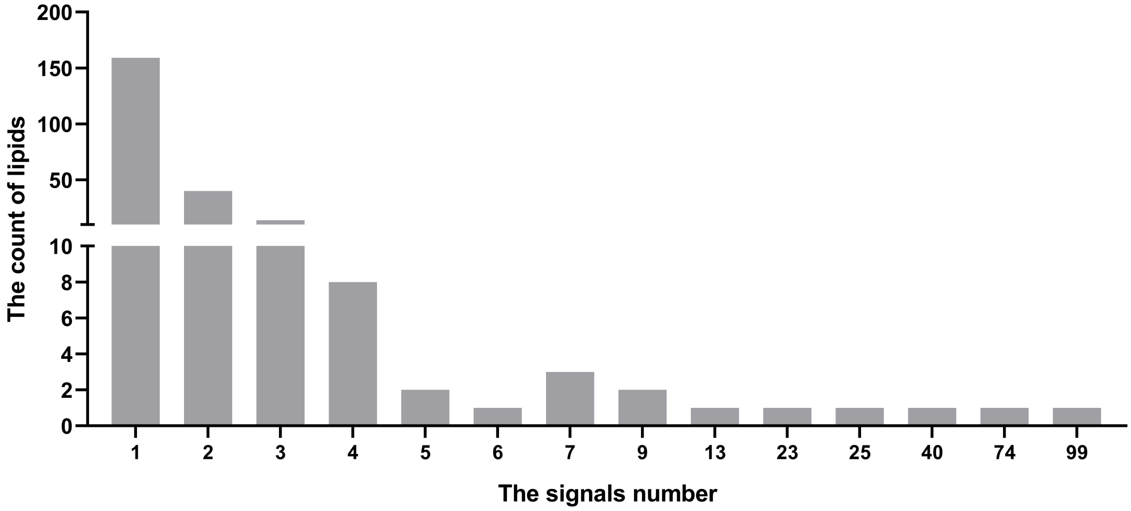


**Figure S4**. Numbers of mGWAS signals for each lipid.

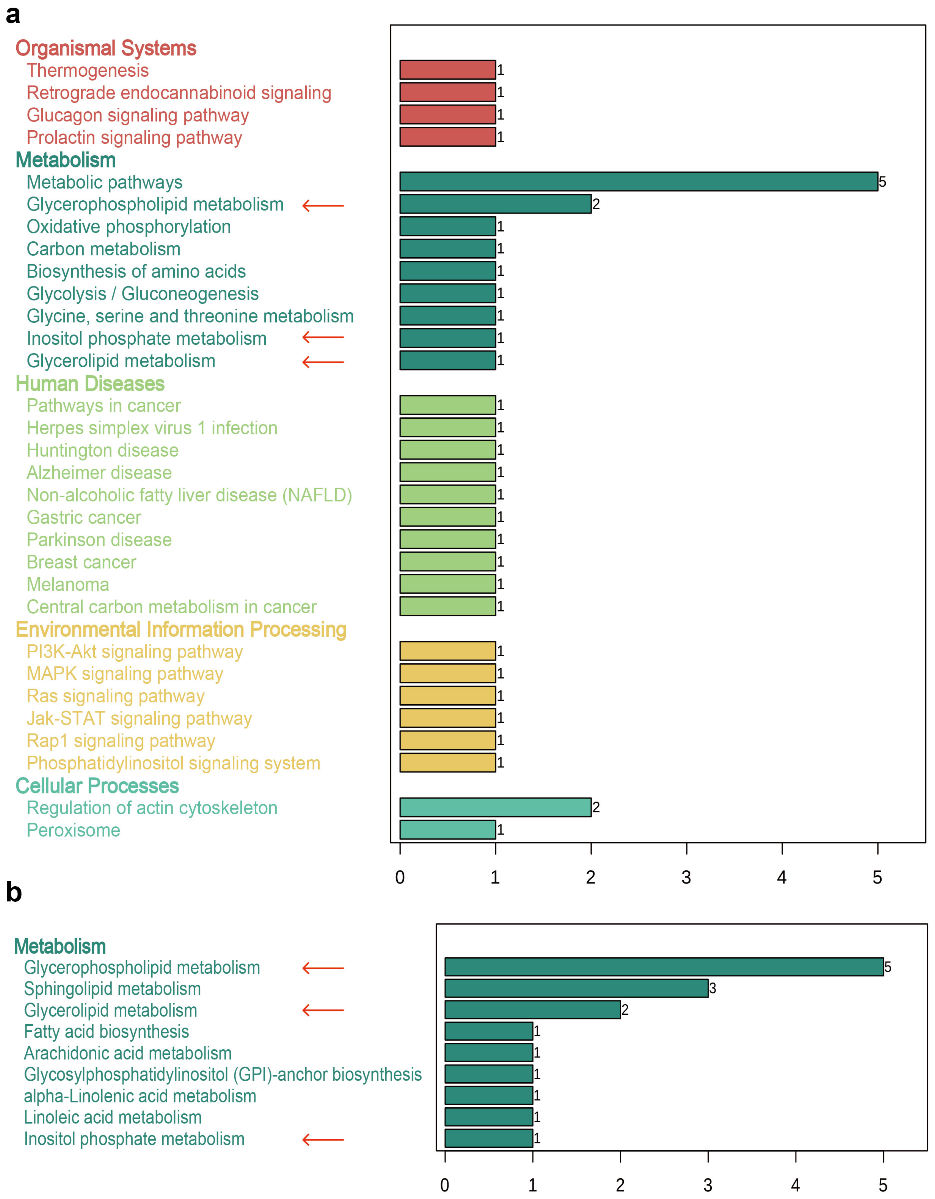


**Figure S5**. KEGG enrichment analysis of candidate genes (**a**) and their associated lipids (**b**). Red arrows indicate co-enriched pathways.

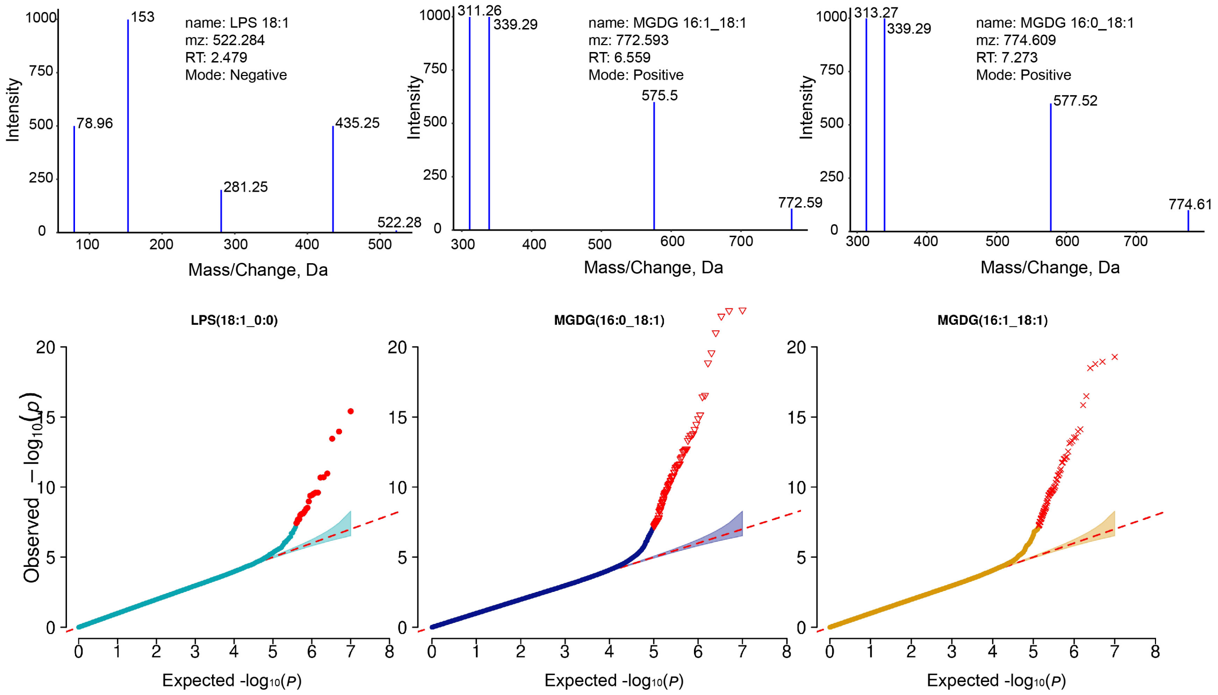


**Figure S6**. EI–MS spectrum and QQ plot of LPS(18:1_0:0), MGDG(16:0_18:1), and MGDG(16:1_18:1).

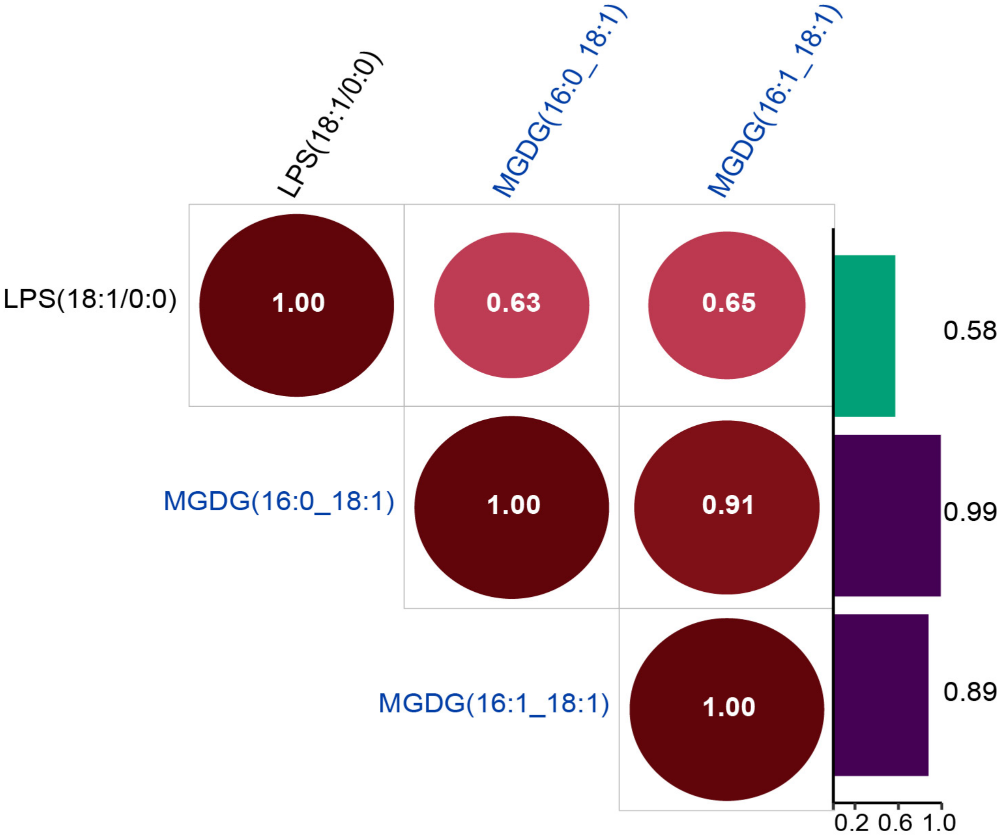


**Figure S7**. Genetic parameters estimation for LPS(18:1_0:0), MGDG(16:0_18:1), and MGDG(16:1_18:1).

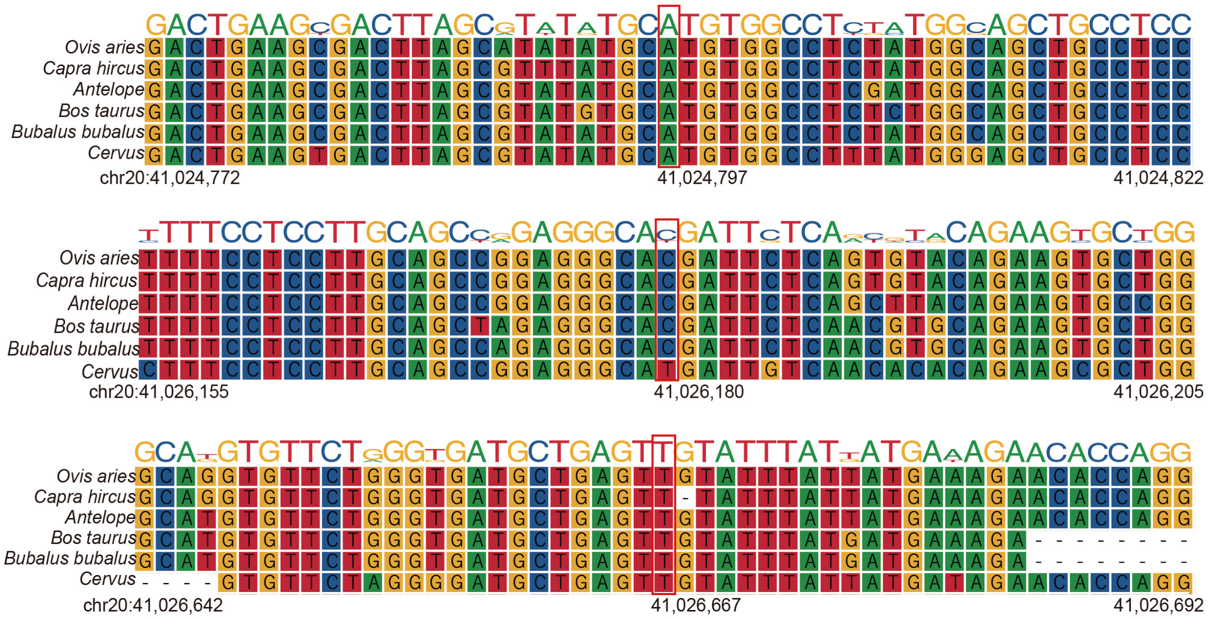


**Figure S8.** Conservation analysis of the lead SNPs for LPS(18:1_0:0), MGDG(16:1_18:1), and MGDG(16:0_18:1) and their flanking sequences across ruminant species.

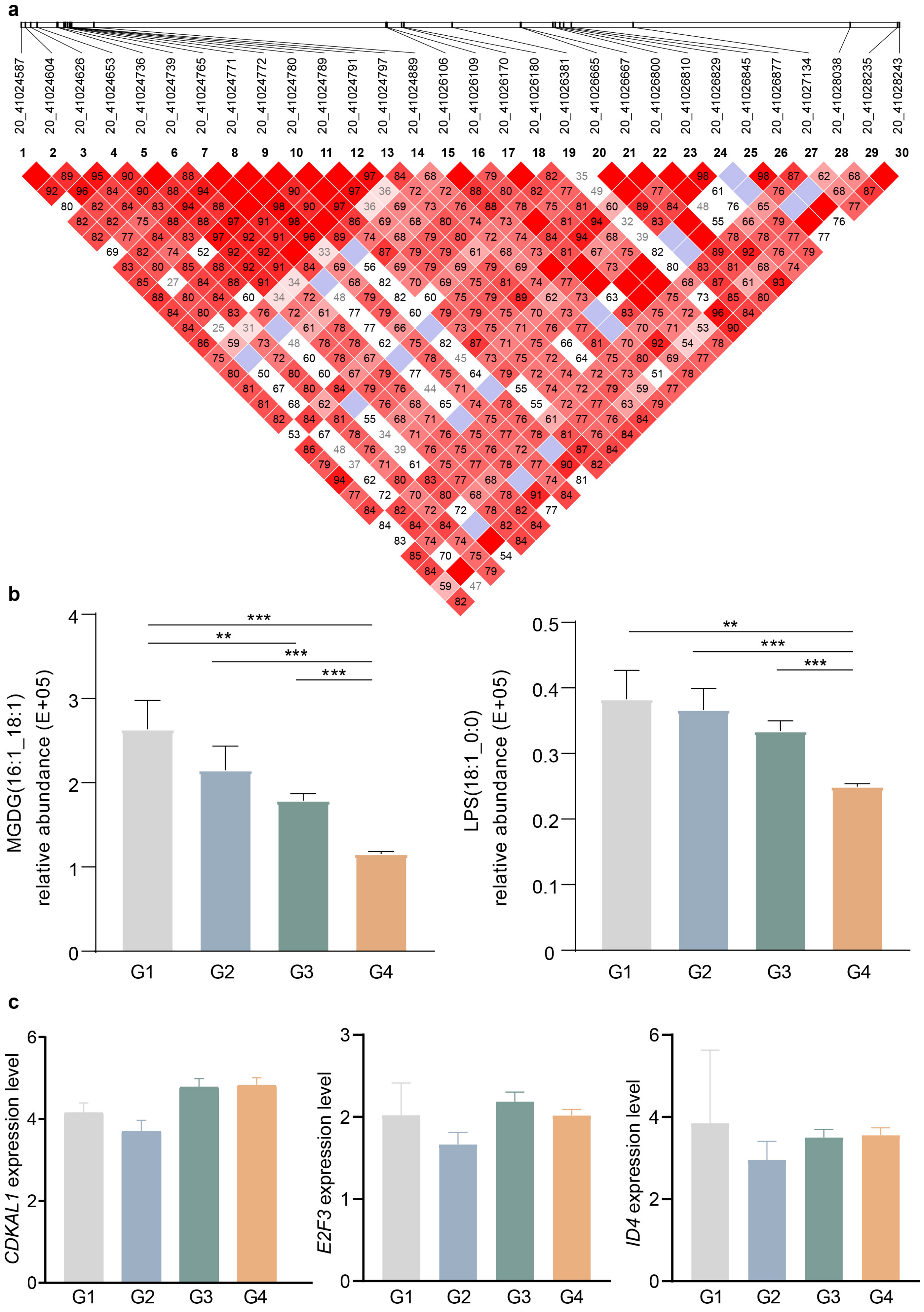


**Figure S9**. Analysis of SNP pairwise linkage disequilibrium (LD) patterns in highly linked regions and effects of combined genotype on lipid abundance and gene expression. (**a**)

Pairwise LD analysis of 30 highly linked SNPs. (**b**) Relative abundance of MGDG(16:1_18:1) and LPS(18:1_0:0) across four genotype combinations. (**c**) Expression levels of three genes within the 1 Mb region surrounding the lead SNP across four genotype combinations. Data are presented as mean ± SEM. **$P < 0.01$, ***$P < 0.001$.

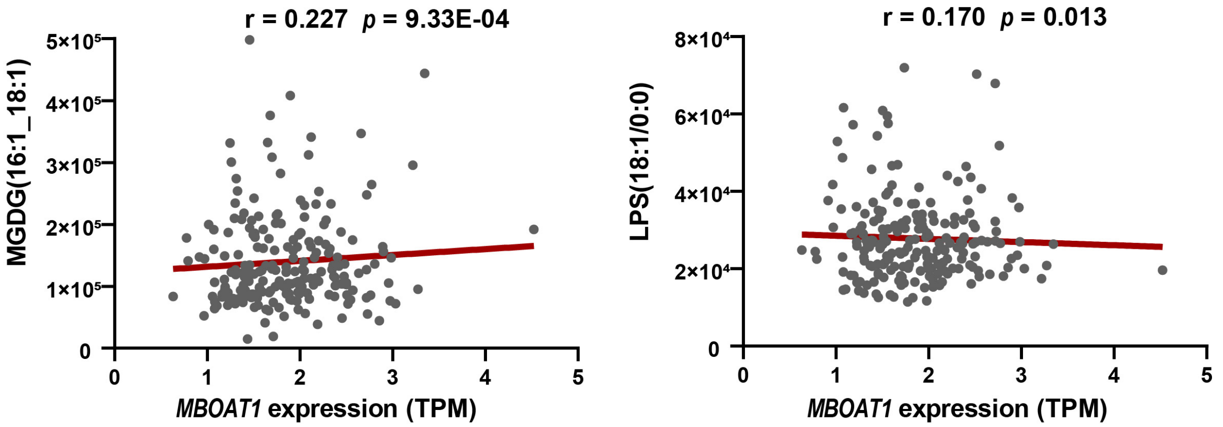


**Figure S10**. Correlation between *MBOAT1* expression and lipids abundance. Data represent Pearson correlation coefficients (r) and the red solid lines indicate linear regression fits. Statistical significance was determined using two-tailed t-tests (n=210).

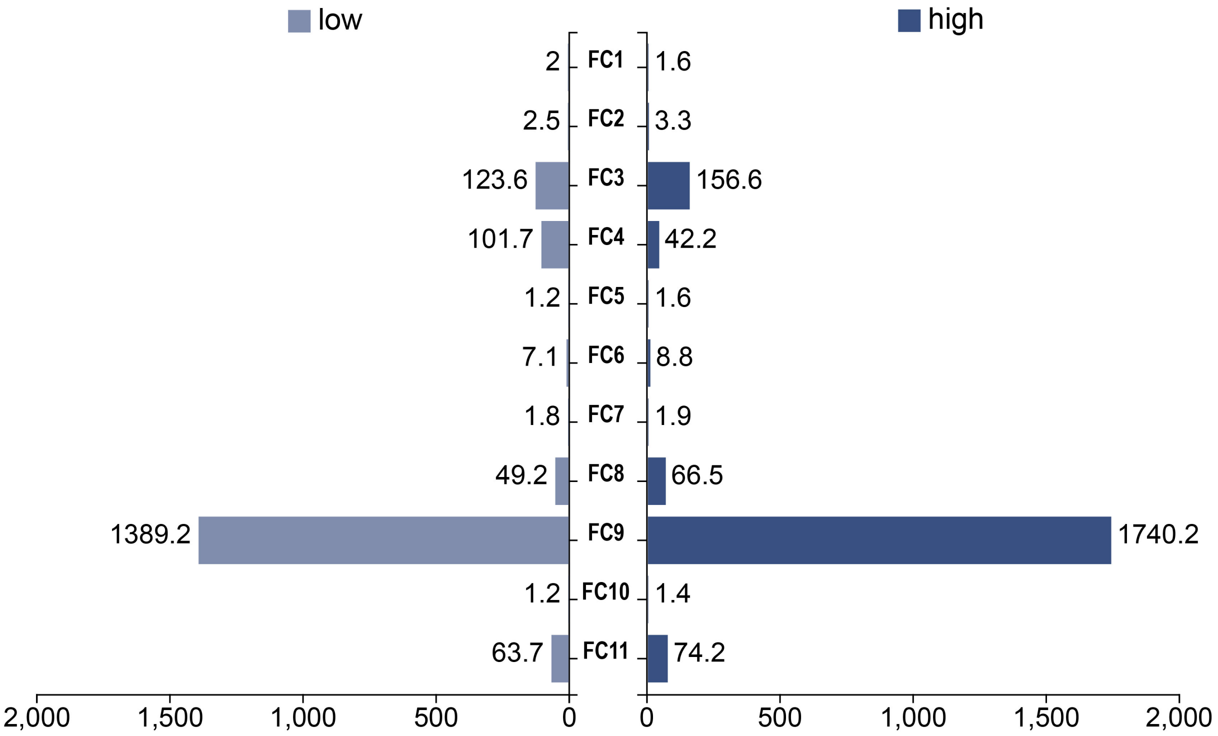


**Figure S11.** Relative odor activity values (rOAVs) of differential flavor compounds in high- and low-abundance cohorts of C18:1-containing MGDG and LPS lipids. Data calculated as mean compound concentration to odor detection threshold ratios (n = 20).

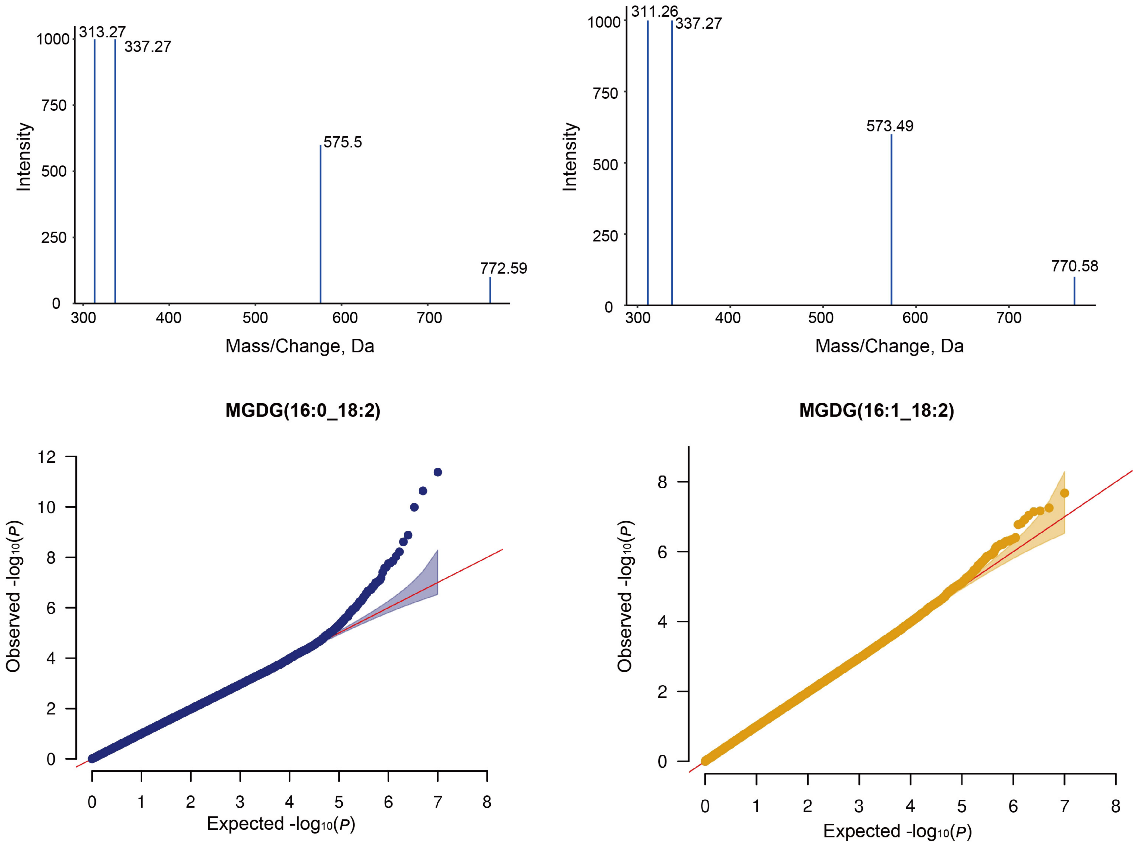


**Figure S12.** EI–MS spectrum and QQ plot of MGDG(16:0_18:2) and MGDG(16:1_18:2).

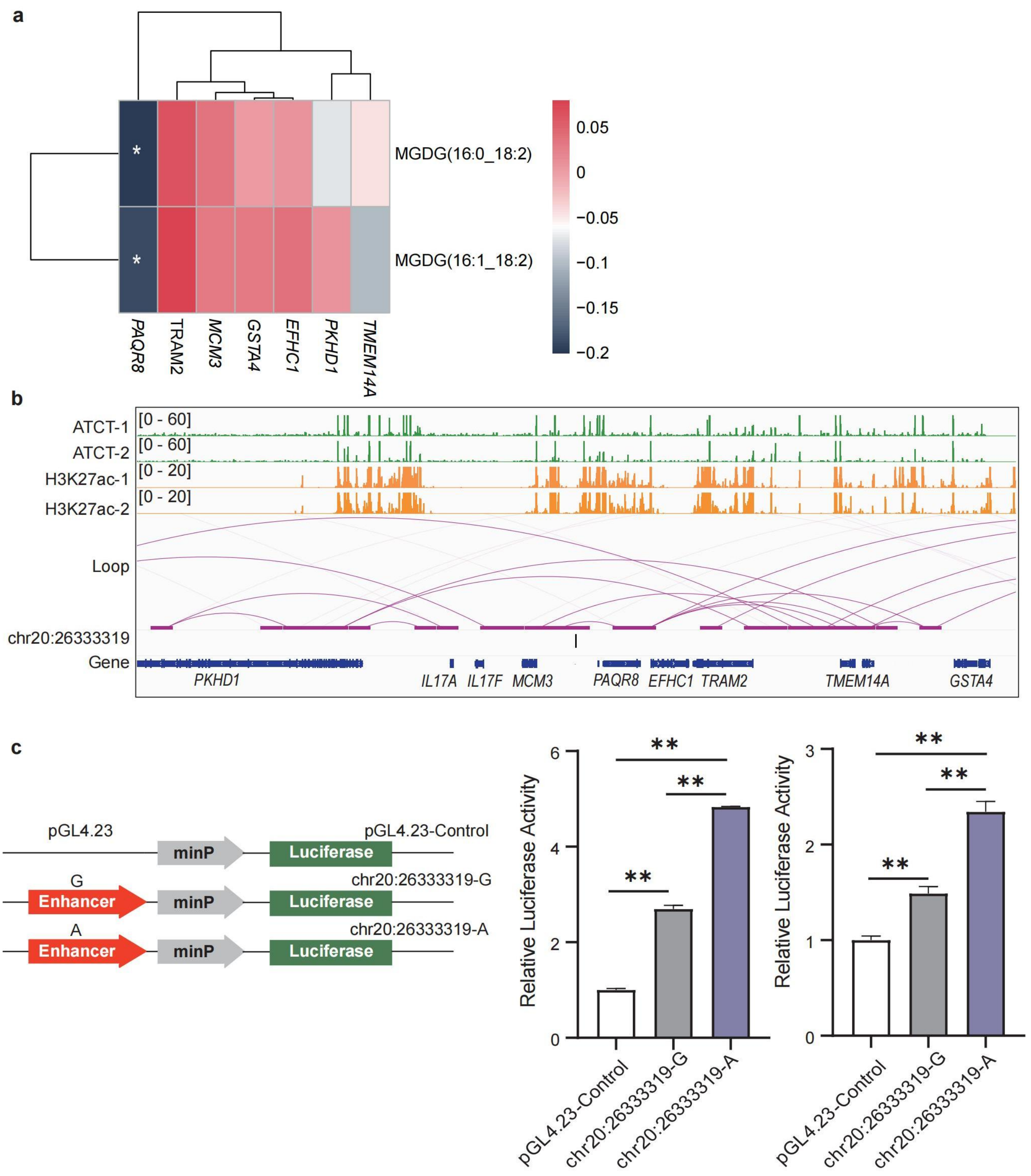


**Figure S13.** Functional analysis of the chr20:26,333,319 locus. (**a**) Correlation matrix of lipid abundance versus expression levels of genes within ±1 Mbp flanking the shared SNP (chr20:26,333,319) (n = 210). Heatmap tiles represent Pearson correlation coefficients (r) with significance thresholds: *$P < 0.05$. Gene expression data derived from RNA-seq (TPM normalization). *IL17A*/*F* expression was not detected in sheep muscle. (**b**) Analysis of chromatin accessibility (ATAC-seq), active enhancer/promoter marks (H3K27ac profiling), and chromatin interactions (Hi-C loops). (**c**) Schematic of luciferase reporter vector design and luciferase activity assays in 293T cells (left) and sheep intramuscular preadipocytes

(right). The chr20:26,333,319 locus exhibited divergent activity changes relative to *PAQR8* gene expression levels following site-directed mutagenesis. Data presented as mean ± SEM. **$P < 0.01$.

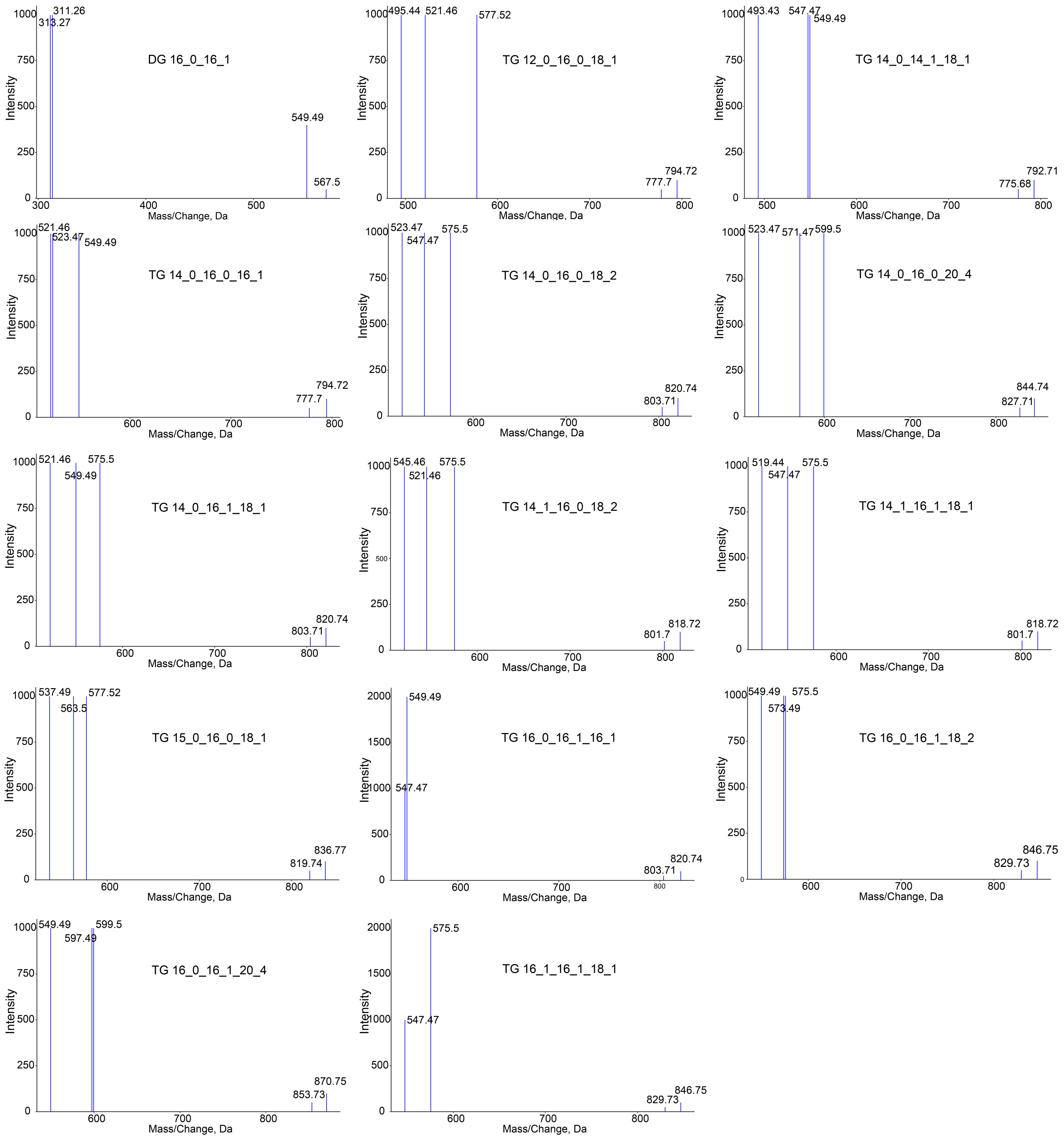


**Figure S14.** EI–MS spectrum of 13 triglycerides and one diglyceride.

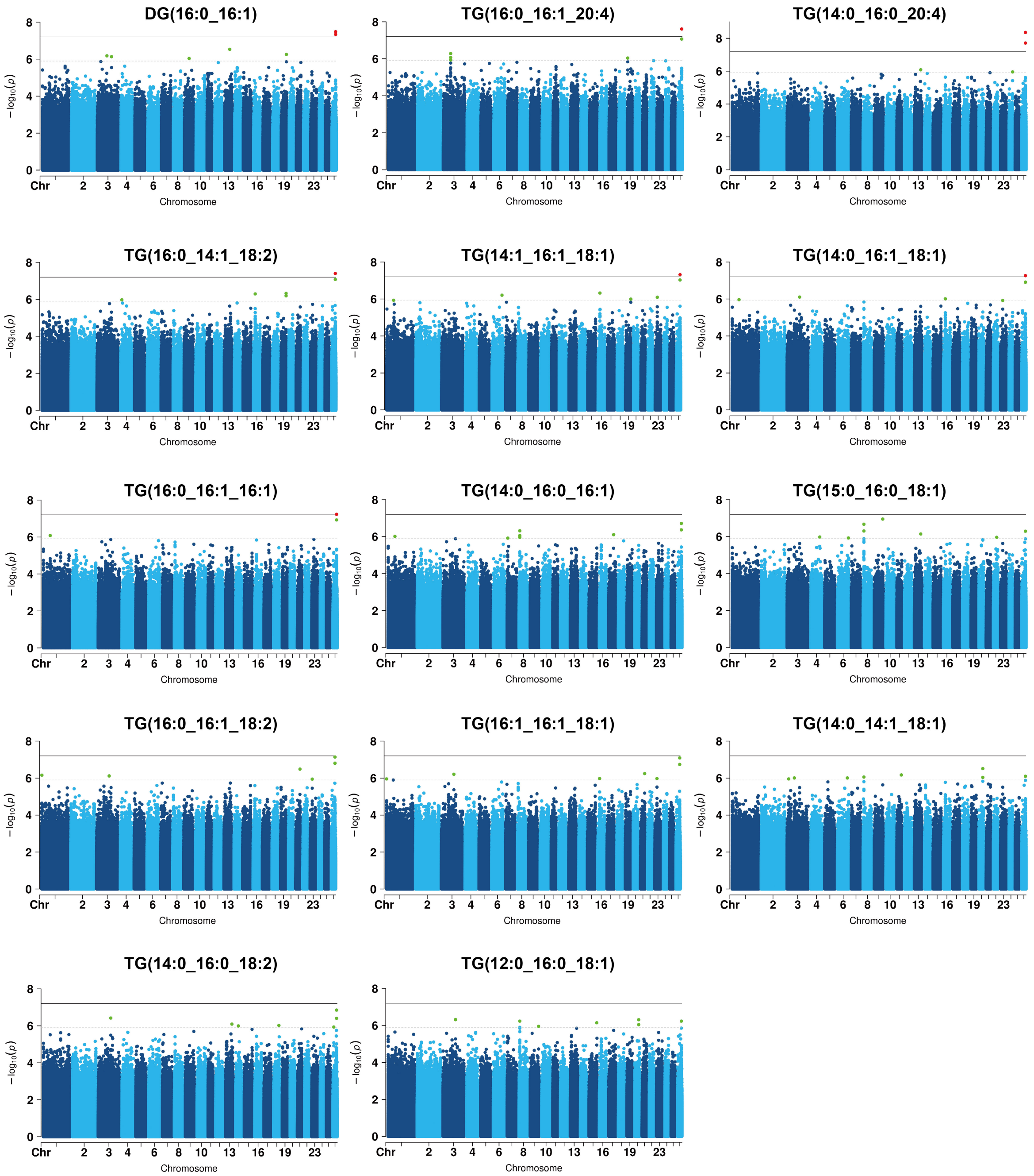


**Figure S15.** Manhattan plot of 13 triglycerides and one diglyceride. Genome-wide significance thresholds were established at $P = 6.26 \times 10^{-8}$ (solid black line) and suggestive significance at $P = 1.25 \times 10^{-6}$ (dashed black line). Red points indicate variants reaching genome-wide significance, while green points represent variants reaching suggestive significance.

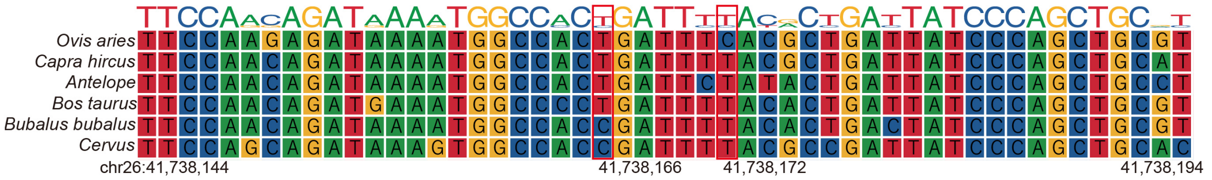


**Figure S16.** Conservation analysis of the SNP associated with 13 TGs and one DG and their flanking sequences across ruminant species.

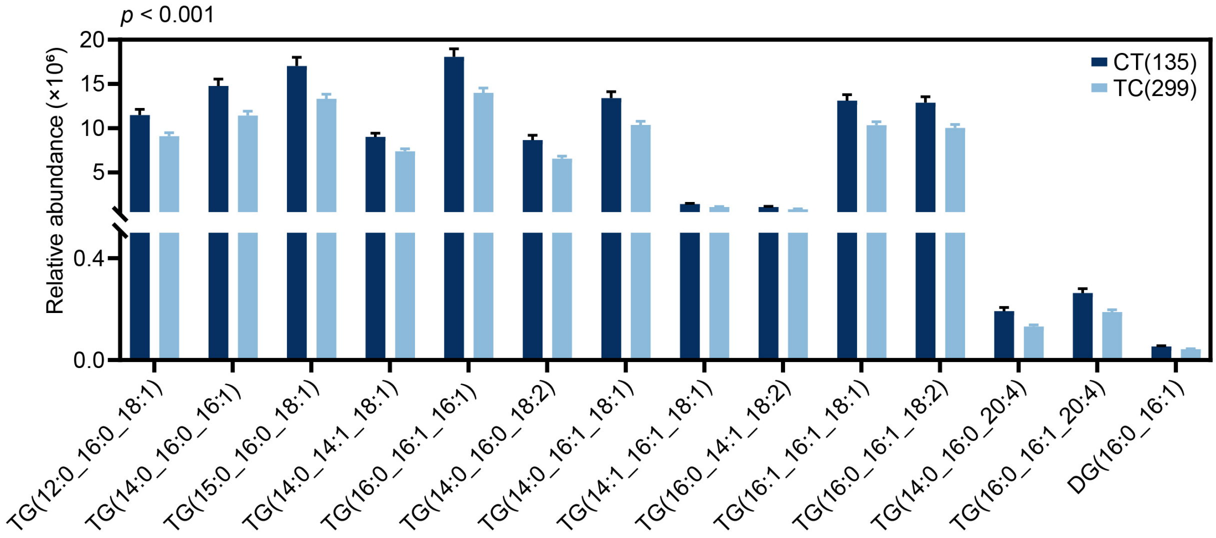


**Figure S17.** Comparative analysis of 14 glycerolipid species (13 triglycerides and 1 diglyceride) between two haplotypes. Data are presented as mean abundance ± SEM, with significance assessed by Student's two-tailed t-tests. All differences were significant at $P < 0.001$.

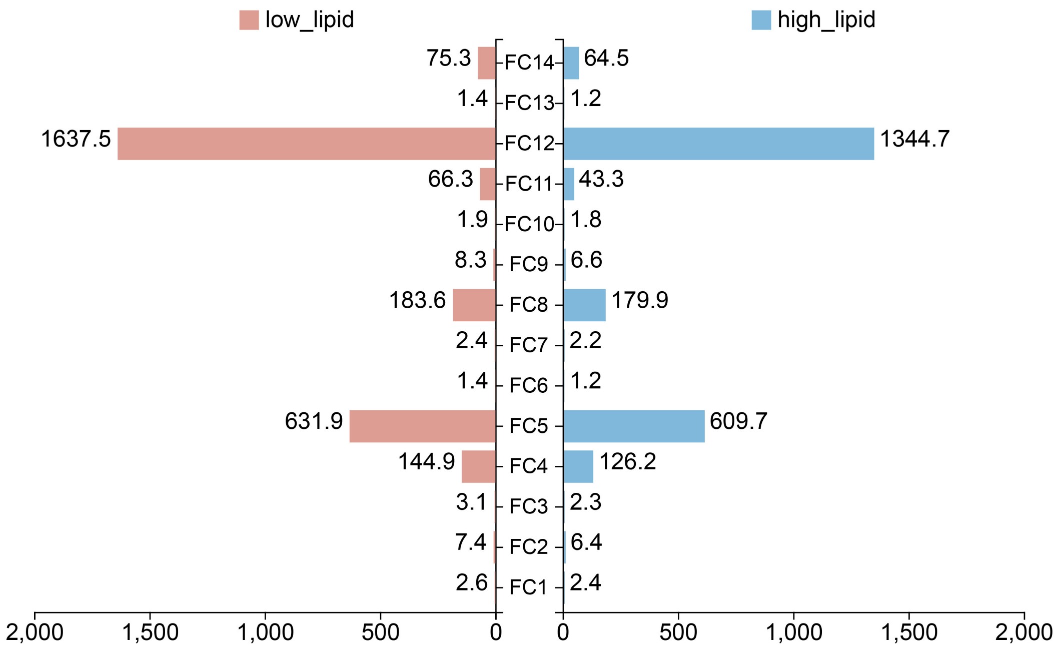


**Figure S18.** Relative odor activity values (rOAVs) of differential flavor compounds in high- and low-abundance cohorts of 14 glycerolipids. Data calculated as mean compound concentration to odor detection threshold ratios (n = 20).

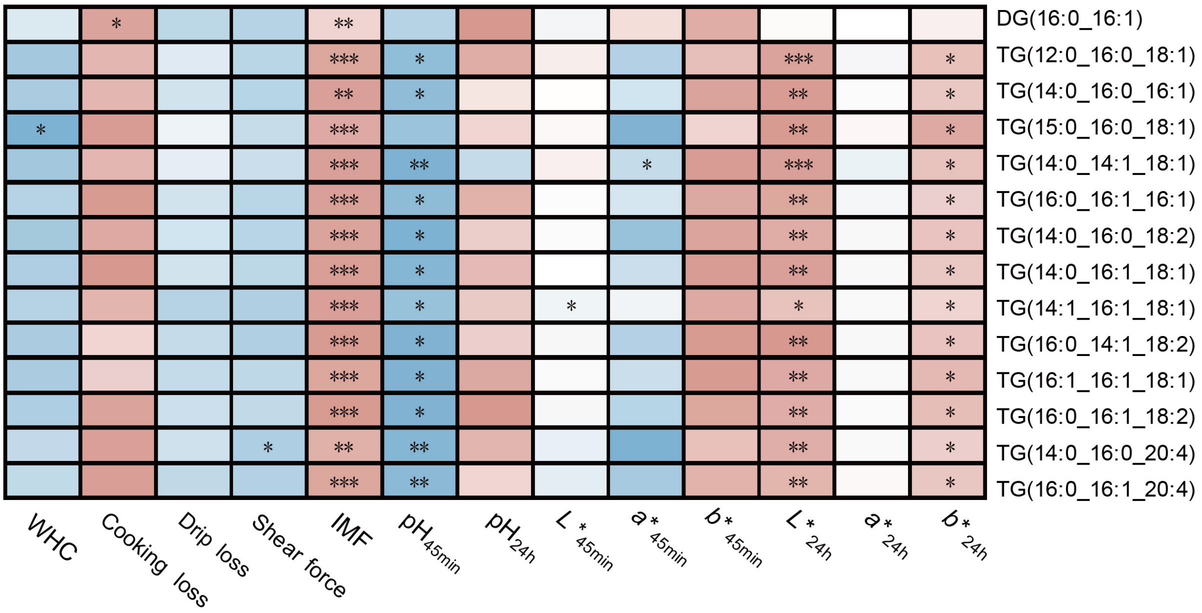


**Figure S19.** Correlation matrix of glycerolipids abundance with meat quality traits. The meat quality indices were assessed as described in Supplementary Methods. $P$*<0.05, $P$**<0.01, $P$***<0.001.

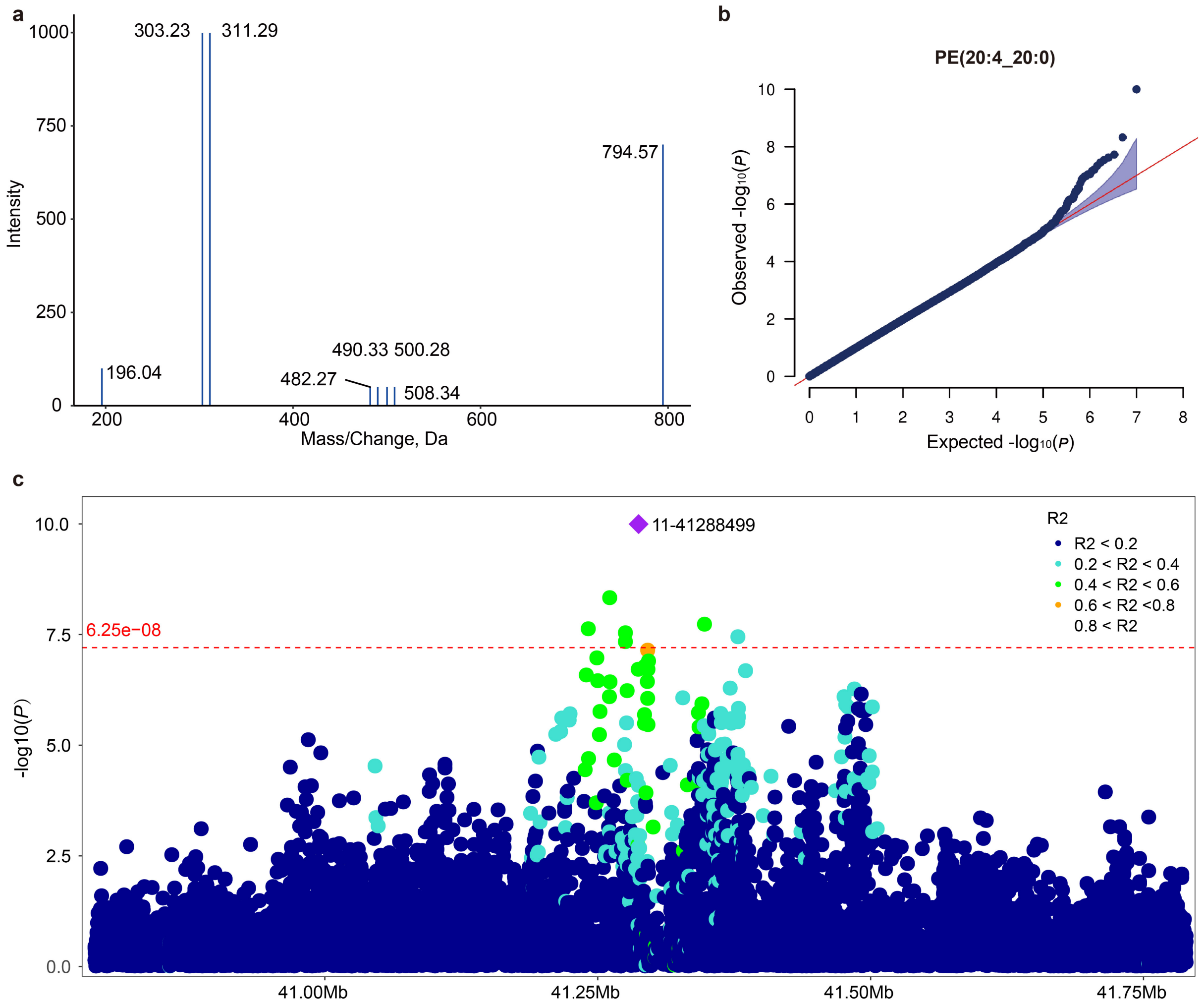


**Figure S20**. Characterization of significant mGWAS signals for PE(20:4_20:0). (**a**) EI-MS spectrum. (**b**) QQ plot of association results. (**c**) Linkage disequilibrium (LD) analysis of SNPs surrounding the lead SNP (± 500 kb).

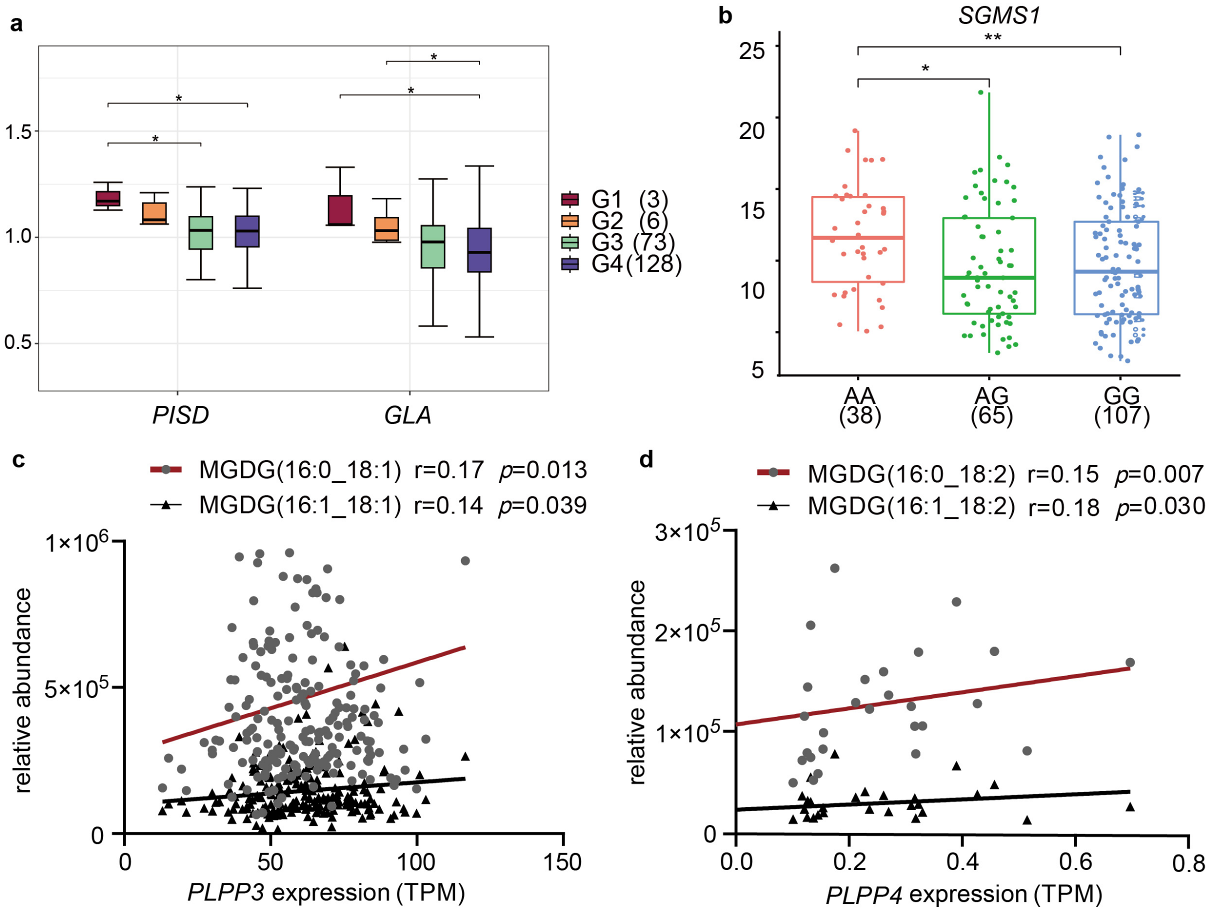


**Figure S21.** Key enzymes driving acyl chain-specific MGDG biosynthesis pathways. (**a**) Genotype-dependent coordinated upregulation of *PISD* and *GLA* for C18:1-MGDG synthesis. (**b**) Differential *SGMS1* expression across genotypes of Chr20:26333319. *P**<0.05, *P***<0.01. (**c**) Significant positive correlation between *PLPP3* and C18:1-MGDG (n = 210). (**d**) Significant positive correlation between *PLPP4* and C18:2-MGDG (n = 27), with samples showing undetectable muscular expression excluded.
